# Recovery of Sesamin, Sesamolin, and Minor Lignans From Sesame Oil Using Solid Support-Free Liquid–Liquid Extraction and Chromatography Techniques and Evaluation of Their Enzymatic Inhibition Properties

**DOI:** 10.3389/fphar.2019.00723

**Published:** 2019-06-28

**Authors:** Dimitris Michailidis, Apostolis Angelis, Nektarios Aligiannis, Sofia Mitakou, Léandros Skaltsounis

**Affiliations:** Faculty of Pharmacy, Department of Pharmacognosy and Natural Products Chemistry, University of Athens, Athens, Greece

**Keywords:** sesame oil, sesamin, sesamolin, liquid–liquid extraction, centrifugal partition extraction, annular centrifugal extraction, centrifugal partition chromatography, collagenase inhibition activity

## Abstract

In this study, an integrated process for the recovery of sesamin and sesamolin, two high added-value lignans of sesame oil (SO) was developed, using synchronous extraction and chromatography techniques. The extraction of SO phenolic content was studied using two different extraction techniques: Annular centrifugal extraction (ACE) and centrifugal partition extraction (CPE). The derived data of each experiment were compared in terms of revealing the yields, time, and solvents consumption showing that CPE is the most effective technique, concerning the solvent consumption. The isolation of lignans was achieved using centrifugal partition chromatography (CPC) both on semi-preparative and preparative scale. The biphasic system used for this purpose consisted of the following solvents: n-Hex/EtOAc/EtOH/H_2_O in proportion 2:3:3:2 (v/v/v/v) and direct recovery of the two major lignans sesamin and sesamolin was achieved. In parallel the CPC analysis resulted in the isolation of four minor lignans of sesame oil, i.e., samin, sesamol, sesaminol, and episesaminol. Structure elucidation of isolated lignans was based on HRMS/MS and NMR experiments. High-performance liquid chromatography (HPLC) was employed for quantitative analysis of the obtained extracts to determine the purity of the isolated compounds as well. The results of this study demonstrated that sesamin and sesamolin were recovered in purity higher than 95%, verifying the effectiveness of the purposed separation methodology. Finally, due to the general application of sesame oil in cosmetic industry, all the pure compounds were evaluated for their tyrosinase, elastase, collagenase, and hyaluronidase inhibition activity.

## Introduction

**Figure 1:**
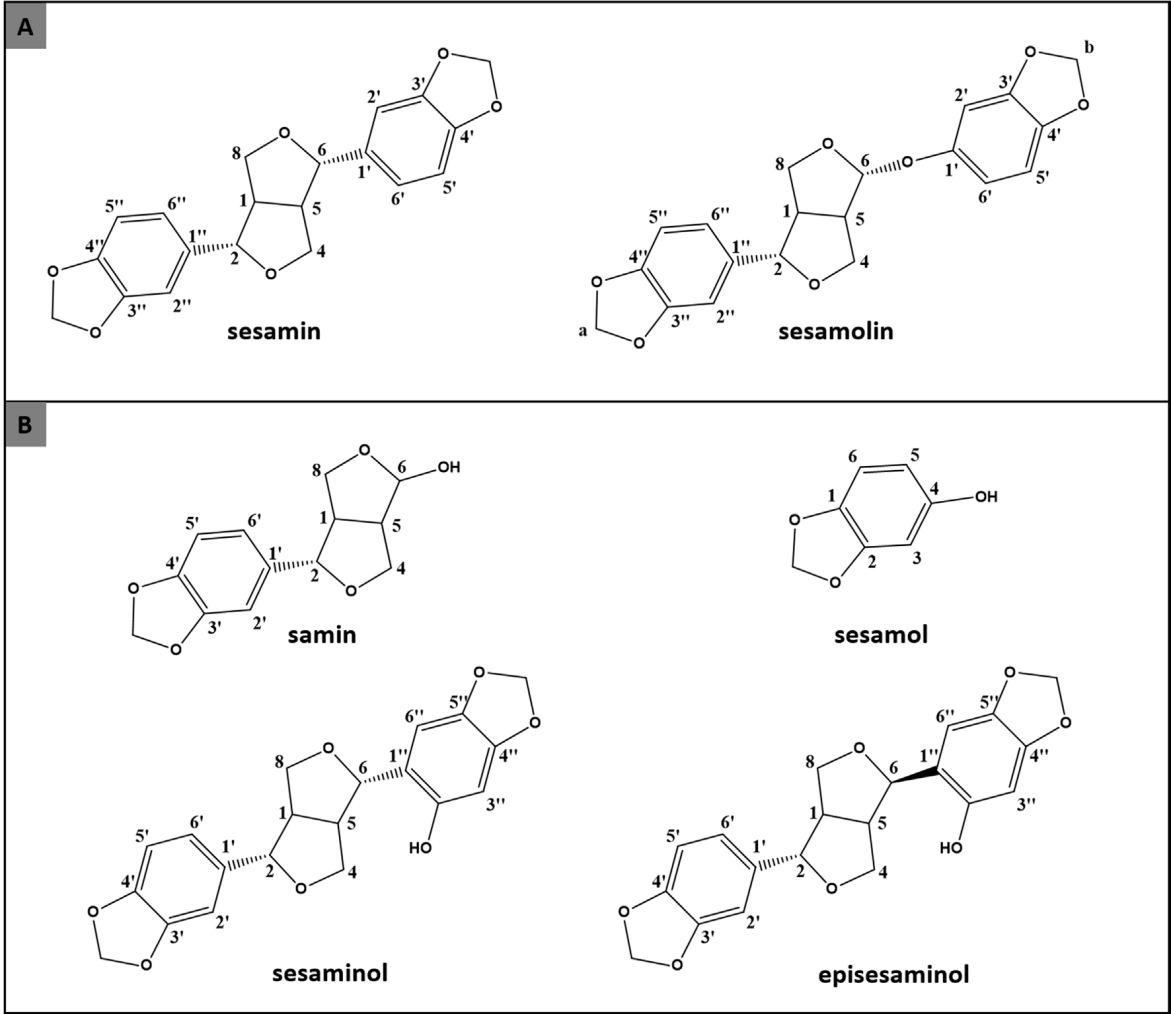
Chemical structures of major (**A**: sesamin and sesamolin) and minor (**B**: samin, sesamol, sesaminol, and episesaminol) lignans isolated from sesame oil.

Sesame oil is a product of high importance, obtained from the seeds of *Sesamum indicum* (Pedaliaceae) and is directly linked to the traditional nutrition of Asian and African people for more than 5,000 years (Zhang et al., 2013; Sarkis et al., 2014). According to Food and Agriculture Organization of the United Nations (FAO) in 2014 the global production of SO exceeded the amount of one and a half million tonne to meet market needs (Food and Agriculture Organization of the United Nations (FAO), 2017). These numbers have attracted scientific interest, leading to the study of the chemical content and bioactivity of SO secondary metabolites (Barbosa et al., 2017; Dossa et al., 2017). Many scientific researches have proved that phenolic compounds of SO have numerous biological activities. Especially sesamin and sesamolin (**Figure 1**), the two major lignans of SO extract, have been tested *in vitro*, *in vivo*, and in clinical studies for numerous activities.

Particularly, *in vivo* experiments have proved the hypocholesterolemic activity of sesamin (Nakai et al., 2003), whereas a clinical study demonstrated positive results against total and LDL-cholesterol on humans, probably synergistic with vitamin E (Penalvo et al., 2006). In addition, this molecule promotes the reduction of fat ratio on human body obviating atherosclerosis and corpulence (Dar and Arumugam, 2013), whereas experiments on gerbils and mice have demonstrated the neuroprotective role of sesamin against cerebral ischemia (Chung et al., 2010; Dar and Arumugam, 2013). Another important activity of this lignan is the anti-inflammatory, *via* the inhibition of delta 5-desaturase (Ohnmacht et al., 2008), an enzyme that is connected with the pro-inflammatory mediators (Obukowicz et al., 1998). When the organism lacks glucose, ketone bodies are used to cover the energy demands. Sesamin is able to increase the ketone body concentration (Anilakumar et al., 2010). This furanofuran lignan also decreases the metabolism of γ-tocopherol and as a result, elevates the concentration of tocopherol (Wu et al., 2009). The antioxidant activity of sesamin and its protective role against damages of alcohol and carbon tetrachloride on liver have been proven as well (Nakai et al., 2003).

Sesamolin, the second major lignan of SO, has also a significant number of biological activities. It induces apoptosis of human lymphoid leukemia Molt 4B cells, inhibits the growth of those cells (Miyahara et al., 2001), and prevents from mutagenic activity of H_2_O_2_ (Grougnet et al., 2012). Sesamolin has also free radical scavenging activity and provides protection against neuronal hypoxia (Park et al., 2010).

Due to the high pharmacological interest of sesamin and sesamolin, there are several works regarding the isolation and purification of these lignans from *Sesamum indicum* materials like sesame seeds, sesame meal, and SO (Lee and Choe, 2006; Wang et al., 2009; Reshma et al., 2010; Dar et al., 2015; Hammann et al., 2015; Jeon et al., 2016). In the literature, silica gel column is referred as separation technique of SO extract, which is mostly applied for laboratory purposes (Lee and Choe, 2006). Moreover, crystallization method is carried out, providing mixture of lignans (Reshma et al., 2010). Another study is based on semi-preparative high-performance liquid chromatography (HPLC), which has limited capacity of sample treatment (Dar et al., 2015). Considering counter-current chromatography, two studies are reported, whereas one of them provide a mixture of sesamin and sesamolin, and the other one is characterized by reduced yield of pure compounds (Wang et al., 2009; Hammann et al., 2015). Only in one study was the centrifugal partition chromatography (CPC) technique applied, but because raw material sesame seed meal was used, while the procedure was more time-consuming, and the results gave lower recovery and lower purity (Jeon et al., 2016). However, there is no previous work for producing these two lignans in high amounts and purity using a low cost and fast methodology. Following this need, our research targeted to develop a novel approach, which could meet the abovementioned parameters.

The following experimental procedure is based on liquid–liquid techniques. Centrifugal partition extraction (CPE) is a solid support-free liquid–liquid extraction technique which is based on the immiscible nature of two phases and the partition of compounds in the formed biphasic system (Berthod et al., 2009; Michel et al., 2011; Ungureanu et al., 2013). This technique is mainly used for rapid fractionations of mixtures, pH zone refining separations as well as for the extraction of liquid nature raw materials, such as edible oils (Ungureanu et al., 2013; Angelis et al., 2017). Low experimental time and solvent consumption rendered it suitable for analytical, preparative, pilot, and industrial scale as well (Hamzaoui et al., 2011; Kumar et al., 2014). Two common extraction methods of CPE are co-current elution and multi-dual mode. In the first method, the biphasic system passes through the column and is separated out of the apparatus. At the second method, column is fed with the stationary phase, and the mobile phase passes through the first. The kinetic nature of the two phases can be changed by the rotation of valve ascending/descending, which sets the inlet of solvents in column. Another extraction technique that was used in this study was annular centrifugal extraction (ACE). This technique can rapidly separate the biphasic systems and is characterized by high mass transfer efficiency per time unit (Jing et al., 2017). The centrifugal force is used, first, to mix the two phases and then to separate them (Duan et al., 2015).

CPC is governed by the same principles of CPE with a difference in the number and the volume of column cells (Hamzaoui et al., 2011). The CPE and CPC columns are metallic cylinders with a number of cells that are proportionally related with theoretical plates. Chromatography column cells are higher in number but smaller in volume than extraction column cells (Goll et al., 2015). One phase (stationary phase) is immobilized by strong centrifugal forces into the column, whereas the other phase (mobile phase) is pumped through the column, separating thus the mixture compounds, on the basis of their partition coefficient (K_D_) (Roullier et al., 2009; Ning et al., 2018). The nature of this technique gives the ability of obtainment the maximum amount of the extract and handling high amounts of sample (Jeon and Kim, 2013; Agalou et al., 2018). Also, this technique permits the use of various polarity solvent systems which result in widening the chromatographic performances (Toribio et al., 2011). Another advantage of CPC is the ability of alternation the mobile phase to stationary during a run, accelerating the recovery of compounds (Sutherland, 2007).

The aim of this study was the development of an effective and capable scaling up process for the treatment of sesame oil and isolation of sesamin and sesamolin in high purity. The primary step was the extraction of phenolic compounds from SO. Two extraction techniques were compared to choose the most advantageous concerning experimental time and extract productivity. The second step was the isolation of sesamin and sesamolin in semi-preparative and preparative scale. The last part of the study was the quantification of the obtained lignans using HPLC-DAD. Also, NMR experiments were used for the identification of the targeted molecules. TLC analysis and HPLC experiments were conducted for the qualification of SO extracts. In parallel, sesamin, sesamolin, and minor compounds isolated from SO were evaluated with enzymatic assays (tyrosinase, elastase, collagenase, and hyaluronidase) for their inhibition activity.

## Materials and Methods

### Reagents

The standards of lignans that were used for the quantitative analysis were purchased from Sigma-Aldrich (Missouri, USA). Also, all the reagents were purchased from Sigma-Aldrich. In detail, for the enzymatic assays mushroom tyrosinase (lyophilized powder, ≥1000 units/mg solid, EC Number: 1.14.18.1), 3,4-dihydroxy-L-phenylalanine, sodium phosphate monobasic, sodium phosphate dibasic, kojic acid, elastase type IV from porcine pancreas (EC Number 254-453-6), N-Succinyl-Ala-Ala-Ala-p-nitroanilide (EC Number 257-823-5), Trizma base reagent grade, elastatinal, collagenase from *Clostridium histolyticum* (released from physiologically active rat pancreatic islets Type V, ≥1 FALGPA units/mg solid, > 125 CDU/mg solid, EC Number: 232-582-9), MMP 2 substrate fluorogenic, chlorexidine, bovine serum albumin (BSA), acetic acid glacial, p-(dimethylamino) benzaldehyde, sodium tetraborate, hyaluronidase (released from bovine testes Type I-S, lyophilized powder, 400–1,000 units/mg solid, EC Number: 3.2.1.35), hyaluronic acid, and tanic acid were purchased also from Sigma-Aldrich. The used solvents for the extraction and separation processes were of analytical grade while those used for UPLC-HRMS analysis were of LC-MS grade. All solvents were supplied from Fisher Scientific (Pennsylvania, USA). TLC analysis was performed on Silica gel 60 F_254_ 20 × 20 cm plates purchased from Merck Millipore (Massachusetts, USA). Sesame oil was provided from HAITOGLOU BROS S.A.

### Apparatus

The extraction of SO phenolic fraction was performed using two different liquid–liquid techniques: CPE and ACE. The CPE experiments were performed on an A-CPC apparatus (Rousselet-Robatel Kromaton, Anonay, France) equipped with a 300-ml capacity extraction column (FCPE300^®^) while solvents pumped with preparative Lab Alliance Series III P300 pumps (Pennsylvania, USA). ACE experiments were performed using laboratory scale BXP012 apparatus (Rousselet-Robatel Kromaton, Anonay, France) with 2.2 ml bowl volume. Basic Verderflex pumps (Castleford, United Kingdom) were used for pumping the solvents through the annular extractor.

Semi-preparative and preparative fractionations of SO phenolic fraction were carried out on an FCPC apparatus (Kromaton, Anonay, France) equipped with a 200-ml capacity chromatographic column (FCPC200^®^) and 1,000-ml capacity chromatographic column (FCPC1000^®^), respectively. Solvents were pumped with preparative Ecom ECP2000 pumps (Prague, Czech Republic). Chromatograms were recorded with a detector UV Flash 14 DAD UV of Ecom (Prague, Czech Republic) and the fractions were collected with a C6-60 Buchi collector (Flawil, Switzerland).

HPLC analysis was performed on a Thermo Finnigan HPLC system (Ontario, Canada) equipped with a SpectraSystem P4000 pump, a SpectraSystem 1000 degasser, a SpectraSystem AS3000 automated injector, and a UV SpectraSystem UV6000LP detector. Data acquisition was controlled by the ChromQuest™ 5.0 software (ThermoScientific™).

Nuclear magnetic resonance spectra were registered on 600 MHz of Bruker AvanceAVIII-600 spectrometer (Karlsruhe, Germany) and was supported by TopSpin software (Bruker). UPLC-HRMS and HRMS/MS analysis was performed on an AQUITY system (Waters) connected with an LTQ Orbitrap Discovery hybrid mass spectrometer (Thermo Scientific) equipped with an ESI source, in negative and positive mode.

For all the enzymatic assays the reader Infinite 200 PRO series (Tecan, Zürich, Switzerland) was used, supported by software Magellan™ (Tecan, Zürich, Switzerland).

### Liquid–Liquid Extraction of SOs’ Lignans

#### Extraction of Lignans Using Laboratory Scale Annular Centrifugal Extractor (BXP012)

Two different experiments were performed using for the extraction the biphasic system SO/Acetonitrile (AcN). In both experiments the rotor speed was set at 3,900 rpm and 200 ml of SO were extracted by using 600 ml of acetonitrile. For the first experiment, the extraction was performed on three successive cycles using 200 ml of AcN in each run (total 600 ml of AcN). AcN (upper phase) and SO (lower phase) were pumped through the apparatus at a flow rate of 8 ml/min for each phase (1/1 ratio of the two phases). The total experiment lasted approximately 1 h and 15 min (∼25 min for each cycle). In the second experiment, the flow rate of AcN was increased at 24 ml/min while the flow rate of SO remained stable at 8 ml/min giving thus a ratio of 1/3 SO/AcN into the extraction bowl. The procedure was accomplished on one single run after 25 min. Samples were collected from each experiment and were analyzed for quantification of the two targeted lignans *via* HPLC technique.

#### Extraction of Lignans Using FCPE300**^®^**

Three extraction runs took place with CPE using multi-dual mode method (Angelis et al., 2017). CPE column was filled with SO (stationary phase) in descending mode, whereas the flow rate and the rotation were set at 20 ml/min and 200 rpm, respectively. Then, AcN was pumped in ascending mode at 10 ml/min and 800 rpm to equilibrate the biphasic system (SO/AcN) inside the column. Stationary phase retention volume was 200 ml and Sf was calculated at 66.6%. Afterward, 240 ml of AcN were collected in 12 fractions of 20 ml. Then, the pumping mode switched to descending and 200 ml of untreated SO replaced the extracted SO with a flow rate of 10 ml/min. The above extraction-recovery cycle was repeated three times of 44 min per run. The extraction solvent (AcN) was evaporated under vacuum at 40°C to dryness to obtain the SO extract.

#### Fractionation of SO Extract Using Semi-Preparative FCPC200**^®^** and Preparative FCPC1000**^®^** Apparatus

##### Solvent System Selection

Seventeen biphasic solvent systems (**Supplementary Table 1**) were created and studied to select the appropriate systems for the CPC separation process. All systems were initially tested regarding the solubility of the extract and settling time and then the suitability of biphasic systems was evaluated by TLC and HPLC-DAD. The procedure was as follows: 10 mg of SO extract were weighed into a 10-ml glass tube, 3 ml of each phase of the pre-equilibrated biphasic solvent systems were added to the sample and shaken vigorously. After equilibration of the biphasic system (t < 1 min), 1 ml of each layer was evaporated to dryness, the residues were diluted in 1 ml of acetonitrile, and analyzed by TLC and HPLC-DAD. The K_D_ values of the target compounds were expressed as the ratio between the peak area in the stationary phase and the peak area in the mobile phase.

##### Semi-Preparative CPC Analysis

The CPC experiment was carried out in elution extrusion mode by using the biphasic system n-Hex/EtOAc/EtOH/H_2_O in proportion 2:3:3:2 (v/v/v/v). Initially, the column was filled with the stationary phase (the upper phase) on descending mode at a flow rate of 10 ml/min and setting the rotation speed at 200 rpm. Then, the rotation speed was maximized at 900 rpm, and the mobile phase was pumped through the column with a flow rate of 5 ml/min on descending mode. After the system equilibration, the retention volume of the stationary phase was calculated at 105 ml giving a high Sf value of 52.5%. Crude SO extract (110 mg) were dissolved in 10 ml of biphasic system and injected into column. In the elution step 350 ml of mobile phase were passed through the stationary phase at a flow rate of 5 ml/min on descending mode. The experiment was completed by passing 200 ml of the stationary phase on descending mode and extruding the column content. All procedures were monitored by UV detector at 255, 275, 280, and 320 nm while the automatic fraction collector was set to collect fractions every 2 min. The total analysis time was 110 min, and finally, 55 fractions of 10 ml were collected.

##### Preparative CPC Analysis

The semi-preparative method was scaled up to preparative column (1,000 ml rotor) adjusting all the experimental parameters to the larger scale. After filling the column with the upper stationary phase (500 rpm and 25 ml/min), the rotation speed was increased to 750 rpm, and the lower phase of the same system (mobile phase) was pumped at 15 ml/min in descending mode to equilibrate the two phases into the column (Sf was calculated at 65%). Then, 900 mg of the extract were diluted in a mixture of the two phases (ratio 7/3 upper phase/lower phase) and injected *via* a 30-ml injection loop. The volume of mobile phase used for the elution step was 1,600 ml while the experiment completed by passing 1,000 ml of the stationary phase in descending mode (extrusion step). The rotation speed and flow rate were kept stable at 750 rpm and 15 ml/min, respectively, during the whole experiment. The total analysis time lasted approximately 170 min, and finally, 130 fractions of 20 ml were collected.

### Quantitative Analyses of Sesamin and Sesamolin in Crude Extracts and CPC Fractions

For the quantitative analysis of the two lignans, the construction of standard calibration curves on HPLC-DAD was necessary. For the separation, a Supelco Analytical (Sigma-Aldrich) HS C18 column, with dimensions 25 × 4.6 mm, 5 μm was used, heated at 40°C. As mobile phase was used in a gradient system consisted of AcN (A) and water (B). The elusion started with 54% of A and reached 79% in 5 min. Then, in 5 min, A reached 83% and during the next 3 min was increased to 95%. The gradient continued for 2 min with A reaching 100%. In 1 min, the solvent system returned to initial conditions and maintained for 4 min. The total running time was 20 min, and the flow rate was set at 1 ml/min. The injection volume was 10 μl. For sesamin, six concentrations were used: 50, 75, 100, 125, 150, and 175 μg/ml. Also, for sesamolin were used: 25, 50, 75, 100, 125, and 150 μg/ml. As internal standard (IS) vanillin was used in a concentration of 10 μg/ml. For the construction of the calibration curves the ratio area of analyte/IS was used. Linearity was evaluated by coefficient of determination, which was over 0.99 for both analytes (**Supplementary Diagrams 1 and 2**).

### Thin Layer Chromatography (TLC), Ultra High-Performance Liquid Chromatography-High Resolution MS/MS (UHPLC-HRMS/MS), and NMR Analysis

TLC plates were developed in dichloromethane (DCM). Plates were observed at 254 nm, 366 nm, and at visible after treatment with a sulfuric vanillin solution (5% w/v in methanol)—H_2_SO_4_ (5% v/v in methanol) and heated at 100°C to 120°C for 1 min.

The phenolic fraction and selected CPC fractions were analyzed using UPLC-HRMS technique. The separation was run in a Fortis C-18 (1.7 µm, 150 × 2.1 mm) column at 40°C. The elution system consisted of water acidified with 0.1% formic acid (A) and acetonitrile (B) in the following gradient mode: 0–2 min 2% B, 2 to 18 min from 2% to 100% B, 18 to 20 min 100% B, 20–21 min from 100% to 2% B, and 21 to 25 min 2% B. The flow rate was set at 0.4 ml/min, and the injection volume was 10 µl. Ionization was achieved in negative and positive ion mode (ESI+ and ESI−) at 350°C. The mass spectrometric parameters were: sheath gas and aux gas flow rate 40 and 10 units, respectively; capillary voltage, 30 V; and tube lens, 100 V for the positive mode and capillary voltage of −20 V and tube lens of −80 V for the negative mode. The mass range was adjusted from 113 to 1,000 m/z.

NMR samples were dissolved in 600 μl of deuterated chloroform (CDCL_3_). All the ^1^H NMR experiments were applied on 600.11 MHz, while ^13^C NMR spectra were acquired at 150.90 MHz. During all the experiments, temperature was set at 300°K. Spectral width of ^1^H NMR was set to 14 ppm, offset to 6.5 ppm, and scans number to 32. Concerning 2-D NMR experiments, proton spectra were registered according to the abovementioned parameters with 12 scans number for COSY, while carbon spectra width set to 240 ppm, offset to 110 ppm, and scans number to 32 and 160 at HSQC and HMBC, respectively.

### Tyrosinase, Elastase, Collagenase, and Hyaluronidase Inhibition Assays

Tyrosinase, elastase, and collagenase assays were applied following the enzymatic methods described by Angelis et al. (2016) with some modifications, while the enzymatic assays for the inhibition of hyaluronidase were conducted as described by Kim et al. (2013), with some modifications. All the enzymatic assays provide the competitive inhibition activity of the compounds. Three concentrations of pure compounds, i.e., 500, 100, and 25 µM (final concentration in the well) were used on the above enzymatic assays. Experiments were performed in triplicates and twice in total while the final DMSO concentrations did not exceed 5% of total volume. The inhibition percentage was calculated by the formula: Inhibition (%) = [((X control − X control’s blank) − (X sample − X sample’s blank))/(X control − X control’s blank)] × 100, where X control is the absorbance or fluoresces of the mixture consisting of buffer, enzyme, sample solvent, and substrate, and X sample is the absorbance or fluoresces of the mixture of buffer, enzyme, sample, or positive control solution and substrate. Blanks contained all the abovementioned components except the enzyme. Concerning tyrosinase, elastase, and collagenase enzymatic assays, the half maximal inhibitory concentration (IC50) of each positive control was used as standard of comparison, while at hyaluronidase enzymatic assay the maximal inhibitory concentration (IC100) was used.

Tyrosinase enzymatic assay: This assay measures the inhibition of the tested samples at the catalytic oxidation of L-DOPA to dopachrome by tyrosinase. Kojic acid (IC50 = 50 µM) was used as positive control. In a 96-well microplate, 80 µl of phosphate-buffered saline (PBS) (1/15 M, pH = 6.8), 40 µl of the tested sample (dissolved in the PBS buffer), and 40 µl of mushroom tyrosinase (100 U/ml) (dissolved in PBS buffer) were mixed and incubated in the dark for 10 min at room temperature. Afterward, 40 µl of 2.5 mM L-DOPA (substrate) dissolved in PBS buffer was added, and the mixture was incubated for 15 min. The 96-well microplate was measured at 475 nm.

Elastase enzymatic assay: Elastase protocol monitors the release of *p*-nitroaniline from N-succinyl-Ala-Ala-Ala-p-nitroanilide that is stimulated by elastase. Elastatinal (IC50 = 0.5 µg/ml) was used as a positive control. In a 96-well microplate, 70 μl of Trizma buffer (50 mM, pH = 7.5), 10 µl of tested sample (dissolved in Trizma buffer), and 5 µl of elastase (0.45 U/ml) (dissolved in Trizma buffer) were mixed and incubated in the dark for 15 min at room temperature. Afterward, 15 μl of 2 mM N-succinyl-Ala-Ala-Ala-p-nitroanilide (substrate) dissolved in Trizma buffer was added, and the mixture was incubated for 30 min at 37°C. The 96-well microplate was measured at 405 nm.

Collagenase enzymatic assay: Collagenase fragmentates the fluorescence molecule MMP2. The inhibition of the enzyme was measured concerning the reduction of the fluorescent intensity that was produced. Chlorhexidine (IC50 = 50 µM) was used as a positive control. In a 96-well dark microplate, 120 µl of Tris-HCl buffer (50 mM, pH = 7.3), 40 μl of tested sample, and 40 µl of collagenase (50 µg/ml) from *C. histolyticum* (dissolved in Tris-HCl buffer) were incubated for 10 min at 37°C avoiding light exposure. Afterward, 40 µl of 50.0 µM MMP2 (substrate) (MCA-Pro-Leu-Ala-Nva-DNP-Dap-Ala-Arg-NH_2_) dissolved in Tris-Cl buffer was added, and the mixture was incubated in dark for 30 min at 37°C. The fluorescent intensity of 96-well microplate was measured at an excitation maximum of 320 nm and an emission maximum of 405 nm.

Hyaluronidase enzymatic assay: The inhibition activity of this enzyme was calculated inversely proportional of the production of *N*-acetyl-d-glucosamine. Tannic acid (IC100 = 800 μΜ) was used as positive control; 100 μl of acetate buffer (0.1 M NaCl, pH = 3.5), 150 μl of tested sample (dissolved in acetate buffer), and 50 μl of hyaluronidase solution 1% w/v (dissolved in acetate buffer) were added in Eppendorfs. Afterward, 100 μl of BSA solution 0.2% w/v (dissolved in ddH_2_O) was added in each Eppendorf and incubated for 20 min at 37°C. Then, 50 μl of hyaluronic acid solution 0.5% w/v (dissolved in ddH_2_O) was added and incubated for 60 min at 37°C; 45 μl from each Eppendorf was transferred in new Eppedorfs containing 10 μl of sodium tetraborate solution 0.8 M (dissolved in ddH_2_O), and heated for 3 min at 100°C and cooled down on ice. In each tube 300 μl of dimethylaminobenzaldehyde (DMAB) solution was added (10% w/v dissolved in 10 N HCl and then dissolved 10 times in acetic acid glacial) and incubated for 20 min at 37°C. Finally, 200 μl from the last Eppendorf was transferred in a 96-well microplate and measured at 586 nm.

## Results and Discussion

### Liquid–Liquid Extraction of Phenolic Compounds from SO

Several extraction processes of phenolic compounds from SO have been reported previously, both in laboratory and large scale (Dachtler et al., 2003; Lee and Choe, 2006; Wang et al., 2009; Reshma et al., 2010; Dar et al., 2015). However, the described experimental procedures consume large amount of solvents (Wang et al., 2009; Reshma et al., 2010), in some cases the sesame oil-solvent ratio is 1:8 (Dar et al., 2015), and overnight experimental tasks are needed (Dachtler et al., 2003; Lee and Choe, 2006; Reshma et al., 2010; Dar et al., 2015). Also, a lot of the proposed procedures have many steps, like solvent extraction, crystallization, and saponification, working in very low (−40°C, 4°C) and high temperatures (70°C), facts leading to long experimental protocols and high energy consumption (Dachtler et al., 2003; Lee and Choe, 2006; Reshma et al., 2010; Dar et al., 2015).

To avoid all the abovementioned disadvantages, during the extraction process of SO phenols, two different liquid–liquid techniques were compared. Both ACE and CPE techniques use the centrifugal force to achieve a fast mixture and separation of the immiscible liquid phases during the extraction process (Xu et al., 2006; Hamzaoui et al., 2011). These two techniques are characterized as green eco-friendly processes due to the low solvent and energy consumption, with industrial applications (Meikrantz et al., 2002; Duan et al., 2005; Hamzaoui et al., 2011).

#### Selection of the Suitable Method for Liquid–Liquid Extraction

The initial step for the liquid–liquid extraction process was the selection of the most suitable solvent system for the quantitative recovery of bioactive ingredients from SO. Taking advantage of the nonpolar oil nature, SO was used as ingredient of the biphasic system. This fact allowed the treatment of large amount of raw material increasing thus the process efficiency (Angelis et al., 2017). More specifically, several systems were created and tested by TLC and HPLC (**Supplementary Table 2**). The results of this analysis demonstrated that the presence of water as a part of the polar phase (systems ES1-ES8) led to the creation of stable emulsion, and thus, in unsuitable biphasic systems. On the other hand the non-aqueous biphasic systems containing mainly acetonitrile, ethanol or methanol (ES11-ES17) resulted in better separation of the two phases. The following HPLC-DAD analysis showed that the addition of butanol in the biphasic systems (ES11-ES14) (**Supplementary Table 3**) resulted in an unsatisfactory recovery of the lignans from the feed oil phase. In contrast to these results the direct extraction of SO with acetonitrile, methanol or ethanol (systems ES15, ES16, and ES17, respectively) led to the better recovery of the targeted compounds. These three systems were tested using triple funnel extraction of SO with the corresponding solvent, and the recovered upper phases were analyzed by TLC (**Supplementary Figure 1**) and HPLC (**Supplementary Table 4**). Both techniques demonstrated that system ES15 (extraction with AcN) is the most effective in receiving the lignan fraction and, thus, was chosen for the liquid–liquid extraction of SO.

##### Liquid–Liquid Extraction Using ACE

ACE is a liquid–liquid extraction technique with numerous advantages, such as high mass transfer coefficient, high interfacial areas, low solvents consumption, and flexible phase ratios (Tamhane et al., 2014). To find the critical parameters for the analytical scale ACE extraction of SO using acetonitrile it was necessary to standardize the solvents flow rate and rotor speed. After several trials, it was found that the flow rate of SO should be lower than 14 ml/min and the rotation of the annular rotor at 3,800 to 4,050 rpm. Under these conditions, the two phases of the biphasic system are mixed and separate rapidly into the extraction bowl eliminating thus the formation of an emulsion that affects the quality of the extraction. The first experiment aimed to check the extraction efficiency using acetonitrile as extraction solvent. For this purpose, 200 ml of SO were extracted with 600 ml of acetonitrile in three successive cycles (200 ml in each cycle). The experiment lasted 75 min and totally 2.02 g of SO extract was obtained. The quantitative HPLC analysis of the obtained extract showed the present of 1.05 g of sesamin and 0.36 g of sesamolin (5.25 mg of sesamin and 1.80 mg of sesamolin per ml of SO) (**Table 1**). To reduce the process time and to increase the efficiency of the method, the ACE extraction was repeated. The main difference from the previous experiment was that the flow rate of ΑcΝ was increased three times (24 ml/min), replacing the triple extraction. Thus, 200 ml of SO was extracted with 600 ml of AcN in a single run and in total time of 25 min. The procedure resulted in the recovery of 1.68 g of extract, which contains 0.84 g of sesamin and 0.29 g of sesamolin (**Table 1**).

**Table 1:** Comparison of two extraction techniques used for the treatment of SO in regard to yield, time, and solvent consumption.

Extraction Technique	SO volume	Extraction Time	Yield, g/200 ml SO	Solvent consumption	Yield sesamin	Yield sesamolin
ACE	i. 200 mL ii. 200 mL	i. 75 min ii 25 min	i. 2.02 ii.1.68	i. 600 mL AcN ii. 600 mL AcN	i. 1.05 g ii. 0.84 g	0.36 g 0.30 g
CPE	a. 200 mL	44 min	1.97	240 mL AcN	1.01 g	0.35 g
	b. 200 mL	44 min	2.01	240 mL AcN	1.03 g	0.36 g
	c. 200 mL	44 min	1.94	240 mL AcN	0.96 g	0.34 g

As a result, it was observed the continuous receiving of lignans but reduced recovery. At these conditions, 4.20 mg of sesamin and 1.50 mg of sesamolin were obtained from each ml of SO. It is important to note that is the first time that ACE technique was applied for the extraction of bioactive compounds, not only from SO, but generally from edible oils.

##### Liquid–Liquid Extraction Using CPE

The experiment was repeated successfully using multi-dual mode method. SO was used as stationary phase while acetonitrile (mobile phase) was pumped through the SO in ascending mode. After passing approximately one column volume (240 ml) of mobile phase, the experiment stopped and the collected fractions (12 fractions of 20 ml) were analyzed by TLC.

**Figure 2:**
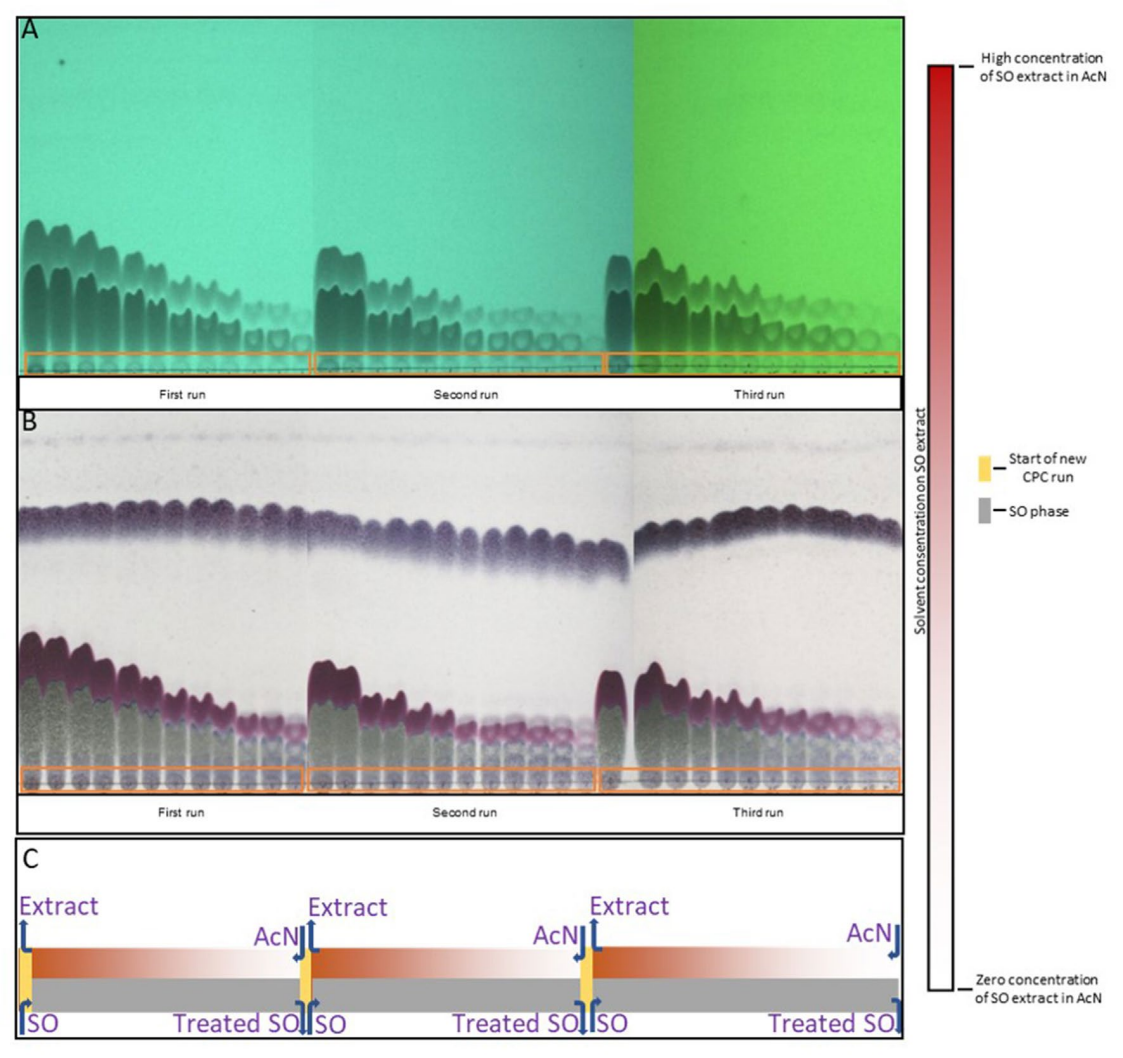
TLC analysis of CPE fractions from three continuous runs in 254 nm **(A)** and in visible sprayed with vanillin solution **(B)**. Schematic presentation of concentration of extraction solvent in lignans during the extraction process **(C)**.

As it is observed in the TLC analysis of CPE fractions (**Figure 2**), the first two fractions are fully enriched in SO extract. Thereafter, the next four fractions are highly concentrated, while the following fractions have a decreasing amount of SO extract. It has to be noted that even the appliance of concentrated spots on the TLC plate, the final fraction provides a negligible amount of the extract. The fact that after 12 fractions we obtained the total amount of SO extract proves the effectiveness of AcN as an extraction solvent. Moreover, it is important to underline the repeatability of CPE technique. The above procedure was repeated two more times by replacing each time the treated SO with the fresh one in descending mode (multi-dual mode process). All the repetitions (three runs) provide exactly the same phenomenon, a total recovery of lignans’ extract after 12 fractions. The fractions of each run were combined, evaporated under vacuum, and weighted, yielding 1.97, 2.01, and 1.94 g, respectively. The quantitative HPLC analysis of the above extracts reveals that sesamin constituted approximately the 50% (1.01, 1.03, and 0.96 g) of total extract, while sesamolin was included also in high amount of approximately 18% (**Table 1**, repetitions a, b, and c).

Overall, the first experiment (i) of ACE provided an adequate extract yield, but with high solvent (ratio SO/AcN 1/3) and time consumption. Concerning the second ACE experiment (ii), the experimental time decreased at 1/3, but the solvent consumption remained the same, while the yield decreased for 17%. Although CPE gave a high amount of extract after reasonable time, the solvent needs decreased almost three times. In detail, after 44 min and with an extraction ratio of 1/1.2 of SO-AcN, CPE technique is able to obtain the total extract of sesame oil. The above seems to lead to the conclusion that CPE is the best extraction solution, because it is efficient on yield, time, and solvent consumption, with high repeatability. It should be highlighted that this technique was applied for the first time, concerning the recovery of lignan fraction from SO.

According to UPLC-HRMS analysis of SO extract, sesamin and sesamolin were detected in the SO extract. Sesamin molecular ion was 355.1176 m/z, and sesamolin molecular ion was 371.1142 m/z in positive mode. Also, other minor lignans as well as fatty acids were detected (**Supplementary Table 5**).

### Separation of Sesamin and Sesamolin from Crude Extracts by CPC

#### Study of the CPC Solvent Systems

Crucial step in the innovated chromatographic process of CPC was to find the biphasic system needed for the separation (**Supplementary Table 1**) and then for the distribution of the target compounds by using TLC analysis. Based on this test, systems CS9, CS10, CS11, CS12, and CS16 were rejected because they do not meet the required specifications (**Supplementary Table 1**) while at systems CS1, CS2, CS14, and CS15, unsatisfactory distributions of sesamin and sesamolin in the TLC chromatograms were observed. Seven biphasic systems (CS3–CS8 and CS17) were further investigated using HPLC to calculate the partition coefficient values (K_D_s) of the target compounds and thus the suitability of the biphasic systems (based on the values of the separation factor α, which follow the rule K_D1_/K_D2_, K_D1_≥K_D2_). The results of this analysis are given in **Table 2**.

**Table 2:** Partition coefficient values (K_D_s) and separation factor (α) of sesamin and sesamolin in seven biphasic systems.

CPC Systems	Partition coefficient of sesamin	Partition coefficient of sesamolin	Separation factor (䌁α) of sesamin and sesamolin
CS3	1.18	0.72	1.63
CS4	1.42	1.76	1.24
CS5	1.20	1.26	1.05
CS6	1.27	0.80	1.59
CS7	1.03	0.37	2.78
CS8	1.15	0.72	1.60
CS17	0.34	0.58	1.71

The study of K_D_s values and separation factors showed that five of the tested biphasic systems (CS3, CS6, CS7, CS8, and CS17) meet the criteria for a satisfactory separation of the target compounds (α > 1.5). Given that the higher value of α enables better separation of the two compounds and the treatment of higher amount of extract, system CS7 (n-Hex/EtOAc/EtOH/H_2_O in proportion 2:3:3:2 v/v/v/v) seems to be the most effective (ခα = 2.78) and thus this system was chosen for the CPC analysis of the SO extract.

#### Purification of Lignans by Semi-Preparative FCPC200**^®^** and the Scale-Up Operation on a Preparative FCPC1000**^®^**

**Figure 3:**
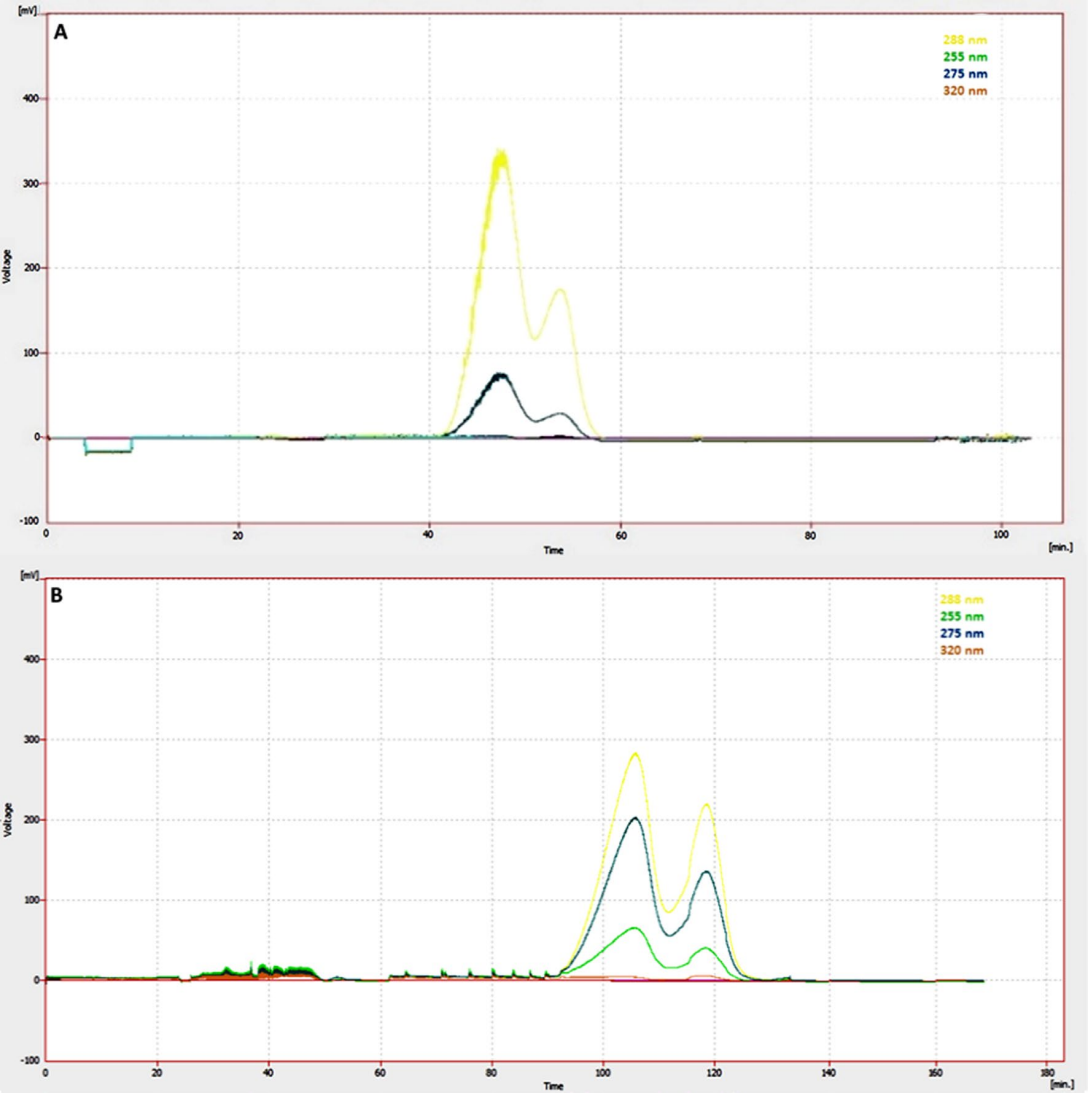
UV Chromatogram (λ = 288, 275, 255, and 320 nm) of semi-preparative elution-extrusion CPC **(A)** in comparison to preparative elution-extrusion CPC **(B)**, indicating the better separation of the two main lignans during the scaling up from semi preparative to preparative mode. Biphasic solvent system: n-Hex/EtOAc/EtOH/H_2_O in proportion 2:3:3:2 (v/v/v/v).

The capability of the selected method (elution–extrusion) and biphasic system (n-Hex/EtOAc/EtOH/H_2_O in proportion 2:3:3:2 v/v/v/v) to efficiently isolate the two major lignans of SO extract was initially tested in semi-preparative column. Except the phenolic part, SO extract contains high amounts of nonpolar fatty compounds, such as glycerides and fatty acids. Due to this nonpolar nature of the extract, the experiment was run in reverse mode by using as stationary phase the upper (nonpolar) phase of the system. This fact stabilizes the fatty compounds at the beginning of the column (due to the close affinity with the nonpolar phase) eliminating thus their co-elution with sesamin and sesamolin. After equilibrating the two phases into the column (Sf = 52.5%), 110 mg of crude SO extract was injected *via* a 10-ml injection loop. The elution step was completed by passing 350 ml of the aqueous mobile phase in descending mode and then the column content was extruded by passing 200 ml of the upper stationary phase also in descending mode. The experiment lasted 115 min while the separation process was monitored by UV at 255, 275, 288, and 320 nm (**Figure 3A**). All resulting fractions (55 fractions of 10 ml) were analyzed using TLC and fractions with similar chemical composition were put together. The result of this analysis was the recovery of 31.6 mg of sesamin and 14.1 mg of sesamolin both in a purity higher than 95% as this was calculated from the quantitative HPLC analysis.

The result obtained from semi-preparative analysis was very promising, and thus separation was scaled up to preparative CPC mode. The scaling up from 200 ml column (semi-preparative) to fivefold larger, 1L CPC column (preparative) can be easily applied, paying particular attention on rotational speed and flow rate, two parameters that affect stationary phase retention (Fumat et al., 2016). After equilibrating the two phases into the column, 900 mg of SO extract was injected. The elution step of the experiment was completed after passing 1,600 ml of aqueous mobile phase in descending mode, and then the column was extruded by pumping 1,000 ml of the upper stationary phase also in descending mode. The CPC procedure was monitored with a UV detector, and the chromatogram (at 255, 275, 288, and 320 nm), presented in **Figure 3B**, shows a recovery of the target compounds according to their K_D_ values in the used biphasic system. By setting the rotational speed at 750 rpm and the flow rate of the eluent at 15 ml/min, the retention factor of stationary phase (Sf) was approx. 65%, much higher than Sf calculated for the semi-preparative CPC experiment (52.5%). Higher retention of the stationary phase led to higher theoretical plate number and thus in better fractionation of the extract. Indeed, the comparison of the CPC chromatograms obtained from semi-preparative and preparative analysis revealed that the preparative process resulted in better separation of the two major lignans (**Figure 3A, B**).

**Figure 4:**
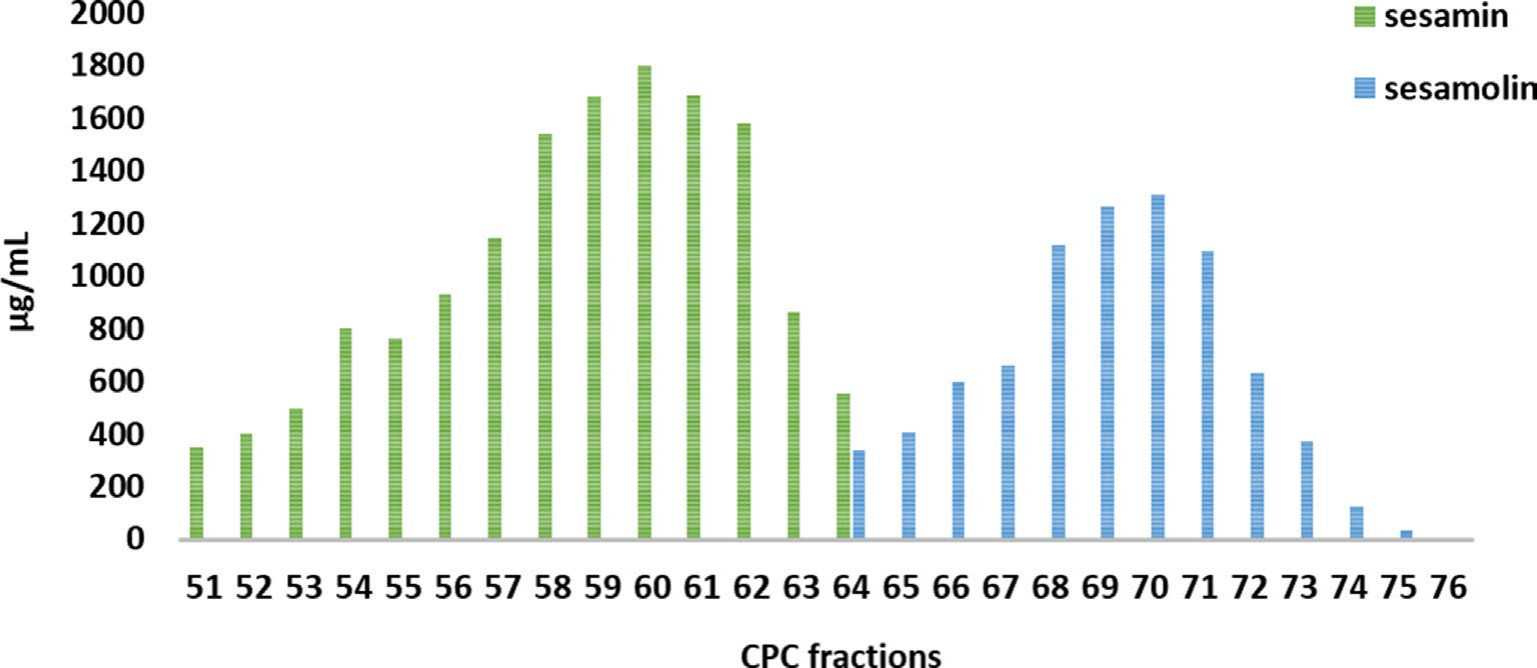
Fractogram obtained from quantification analysis of preparative CPC fractions 51 to 76.

The preparative CPC process lasted 170 min while fraction collector was set to collect 20-ml fractions (total 130 fractions). All fractions were analyzed by TLC to check the quality of the separation. The analysis showed that the lignans were recovered during the elution step of the experiment while the fatty compounds were collected in the last fractions of the experiment during the extrusion of the column content. The fractions containing sesamin (fractions 51–64) and sesamolin (fractions 64–75) were subjected to quantitative HPLC analysis. The result of this analysis (presented as fractogram in **Figure 4**) showed that fractions 51 to 63 and 65 to 75 contain only sesamin and sesamolin, respectively, whereas only one fraction (64) contains a mixture of both compounds. Sesamin fractions were pooled and evaporated to dryness, yielding 276.07 mg, whereas the combined sesamolin fractions yielded 138.15 mg. The following UPLC-HRMS, NMR, and quantitative HPLC analysis of combined fractions showed that both sesamin and sesamolin were isolated in high purity (>95%) and good recovery (61.3% and 87.7% of the total amount of their SO extract content, respectively) verifying the efficient separation of these two bioactive compounds by using the proposed preparative elution–extrusion CPC method.

Except the isolation of the two major lignans, the preparative CPC analysis led to the additional separation of four minor lignans of SO extract, i.e., samin, sesamol, sesaminol, and episesaminol (**Figure 1**). In more details, fractions 13–18 (4.3 mg) samin, fractions 22–31 (9.4 mg) sesamol while fractions 35–39 (8.2 mg) and fractions 40–49 (6.6 mg) contained a mixture of sesaminol and episesaminol in ratios of approximately 85/15 and 45/55, respectively. It is important to note that samin and sesamol were recovered in one step separation procedure in high purity as this was determined by ^1^H-NMR analysis (**Supplementary Figures 2–6**). The structure elucidation of the isolated compounds was achieved by studying HRMS/MS and NMR (1D and 2D) spectra and verified by comparison of the experimental data with the corresponding bibliographic data (Yamauchi et al., 2000; Dachtler et al., 2003; Kuo et al., 2011; Xia et al., 2016; Liu et al., 2018a; Liu et al., 2018b). Experimental data of ^1^H and ^13^C NMR of the isolated compounds are referred to at **Supplementary Table 6**.

### Tyrosinase, Elastase, Collagenase, and Hyaluronidase Inhibition Activity of SO Compounds

All isolated lignans were evaluated for their tyrosinase, elastase, collagenase, and hyaluronidase inhibitory activities. For all the enzymatic assays, the IC50 of positive controls was used, with only exception being the hyaluronidase assay where the positive control was used at the IC100 concentration (see experimental part).

**Figure 5:**
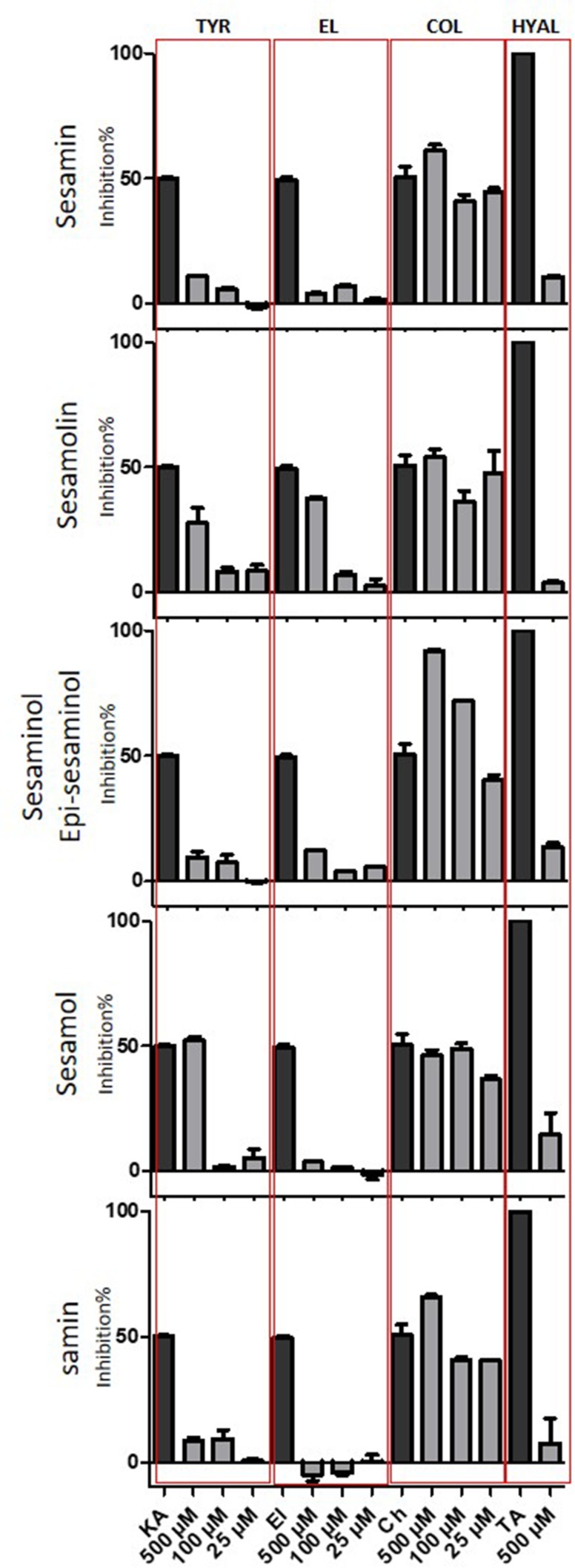
Tyrosinase, elastase, collagenase, and hyaluronidase inhibition activity of isolated compounds. Tested concentration for tyrosinase, elastase, and collagenase inhibition: 500, 100, and 25 µM. Tested concentration for hyaluronidase inhibition: 500 µM. Positive control for tyrosinase: Kojic acid (KA), Positive control for elastase: Elastatinal (El), Positive control for collagenase: Chlorexidine (Ch), Positive control for hyaluronidase: Tanic acid (TA).

The tyrosinase inhibition assay showed that sesamol and sesamolin are able to inhibit the enzyme activity, in contrast to sesamin, samin, sesaminol, and episesaminol. In detail, sesamol exhibited an important inhibition activity at 500 μΜ (52.34%), while no activity was present at doses of 100 and 25 μΜ. Sesamolin presented moderate anti-tyrosinase activity at 500 μΜ (27.78%) and weak activity at 100 and 25 μΜ (**Figure 5**). These results are in agreement with literature data reporting the potent anti-tyrosinase activity of sesamol and sesamolin (Srisayam et al., 2017). The above results demonstrate a potent correlation between the structure of tested compounds and the anti-tyrosinase activity. Although sesamin, sesamolin, sesaminol, and episesaminol are structurally related compounds, only sesamolin inhibited the tyrosinase activity, showing that sesamol moiety seems necessary for the enzyme inhibition.

All tasted compounds expressed important activity on collagenase assay. The sesaminol/epi-sesaminol mixture revealed the highest anti-collagenase activity with inhibition values of 91.99% at 500 μΜ, 71.94% at 100 μΜ, and 40.36% at 25 μΜ. Sesamin presented moderate anti-collagenase activity with inhibition value of 61.16% at 500 μΜ, 40.77% at 100 μΜ, and 44.71% at 25 μΜ. Samin revealed anti-collagenase activity with inhibition values of 65.66% at 500 μΜ, 40.65% at 100 μΜ, and 40.33% at 25 μΜ. Sesamolin also presented moderate activity with inhibition value of 54.05% at 500 μΜ, 36.20% at 100 μΜ, and 47.83% at 25 μΜ, while sesamol revealed the lowest anti-collagenase activity with inhibition values of 46.19% at 500 μΜ, 48.20% at 100 μΜ, and 36.57% at 25 μΜ (**Figure 5**). To our knowledge, this is the first report connecting SO lignans with collagenase activity.

Regarding the elastase and hyaluronidase inhibition assays, all the tested compounds were found to be non-effective compared with the positive controls. The only exception was sesamolin, which presents a moderate anti-elastase activity at the highest dose of 500 μΜ with inhibition value of 37.24% (**Figure 5**).

## Conclusion

This study constitutes a holistic procedure for the swift isolation of sesamin and sesamolin in high purity, using techniques with scale up to pilot and industrial prospects. Two different approaches were used for the extraction of SO lignans based on innovative liquid–liquid techniques, ACE and CPE, to obtain both sesamin and sesamolin in high amounts. However, CPE needs almost one third of solvent volume that was required from ACE to obtain the total extract. The green characteristic is not the only advantage of CPE. Also, this procedure is less time-consuming. CPC, as the superior liquid–liquid solid support-free technique, can treat the SO extract, giving high recovery of sesamin and sesamolin with purity over 95% with the minimum time consumption. The ability of CPC technique to analyze high portions of sample has as a result the isolation of other minor compounds from the SO extract in high purity. Obtaining compounds in pure form permitted the realization of enzymatic assays. As a result, significant anti-collagenase activity was observed from all the isolated molecules.

## Data Availability Statement

All datasets (generated/analyzed) for this study are included in the manuscript and the supplementary files.

## Author Contributions

DM, AA and LS conceived and designed the experiments. DM and AA performed the phytochemical and analytical experiments and wrote the paper. DM and SM performed the biological experiments and analyzed the data. ΝΑ, SM, and LS critically revised the manuscript. All authors read and approved the final manuscript.

## Funding

The research work was supported by the Hellenic Foundation for Research and Innovation (HFRI) and the General Secretariat for Research and Technology (GSRT), under the HFRI PhD Fellowship grant (GA. 14498). The present work was co-funded by the European Union (ERDF) and Greek national funds through the Operational Program “Competitiveness, Entrepreneurship and Innovation,” under the call “STRENGTHENING RESEARCH AND INNOVATION INFRASTRUCTURES” (project code: 5002803).

This work has benefited from HAITOGLOU BROS S.A. with its kind offer of sesame oil.

## Conflict of Interest Statement

The authors declare that the research was conducted in the absence of any commercial or financial relationships that could be construed as a potential conflict of interest.
