# Supplementary material for: Recovery of Sesamin, Sesamolin, and Minor Lignans From Sesame Oil Using Solid Support-Free Liquid–Liquid Extraction and Chromatography Techniques and Evaluation of Their Enzymatic Inhibition Properties
Source: Front Pharmacol. 2019 Jun 28;10:723. doi: 10.3389/fphar.2019.00723 (PMC6610769; doi:10.3389/fphar.2019.00723)
Supplement: Supplementary file 1 [file DataSheet_1.pdf]

Supplementary Material

1 Supplementary Figures and Tables

1.1 Supplementary Figures

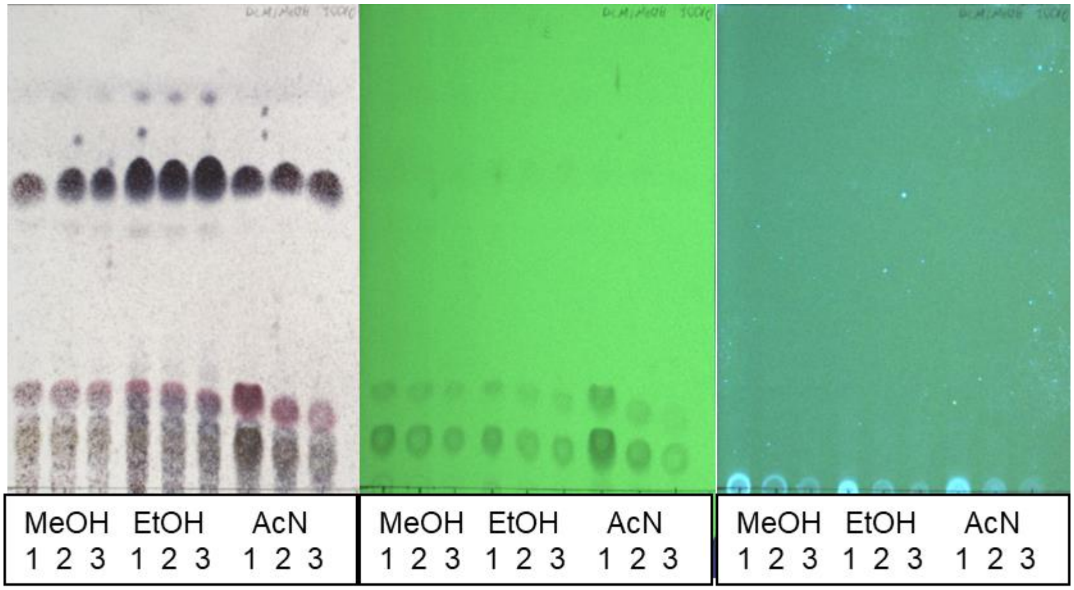

Supplementary Figure 1: TLC of systems ES15-ES17 in visible sprayed with vanillin solution (left photo), in 254 nm (middle photo) and in 366 nm (right photo).

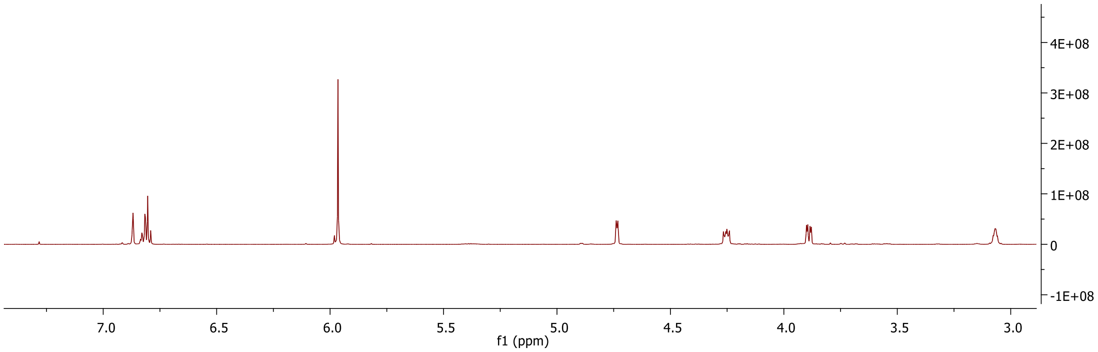

Supplementary Figure 2: a) ^{1}H NMR spectra of sesamin in $CDCl_3$.

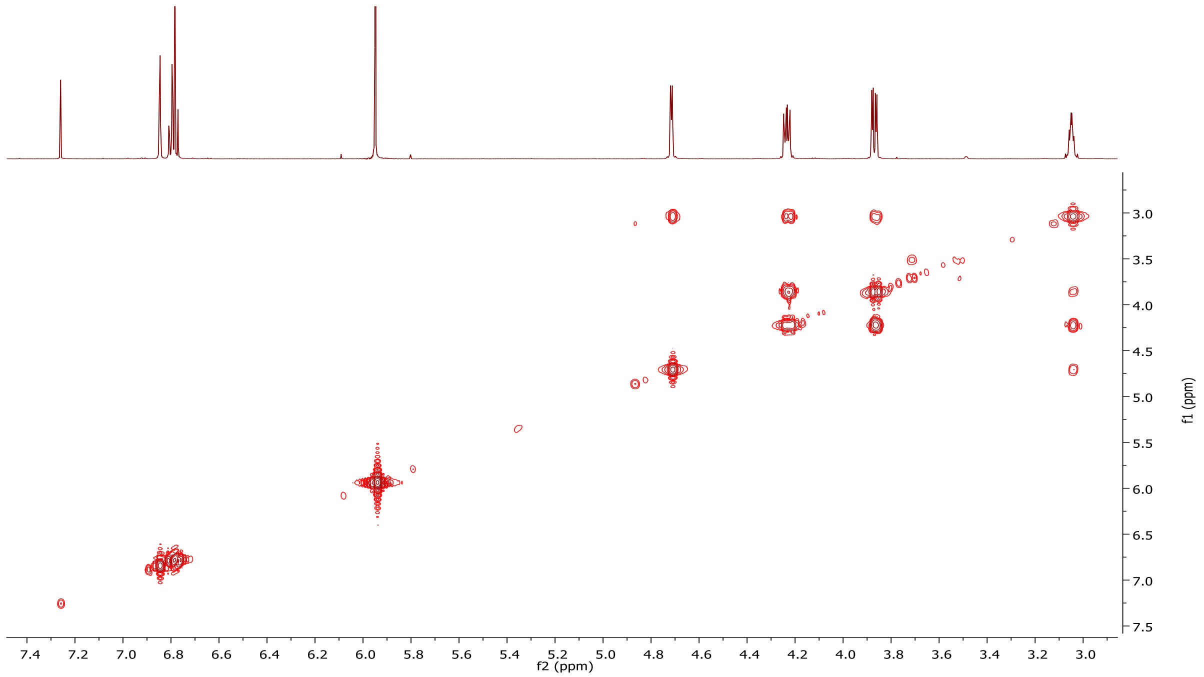

Supplementary Figure 2: b) 2D COSY NMR spectra of sesamin in $CDCl_3$.

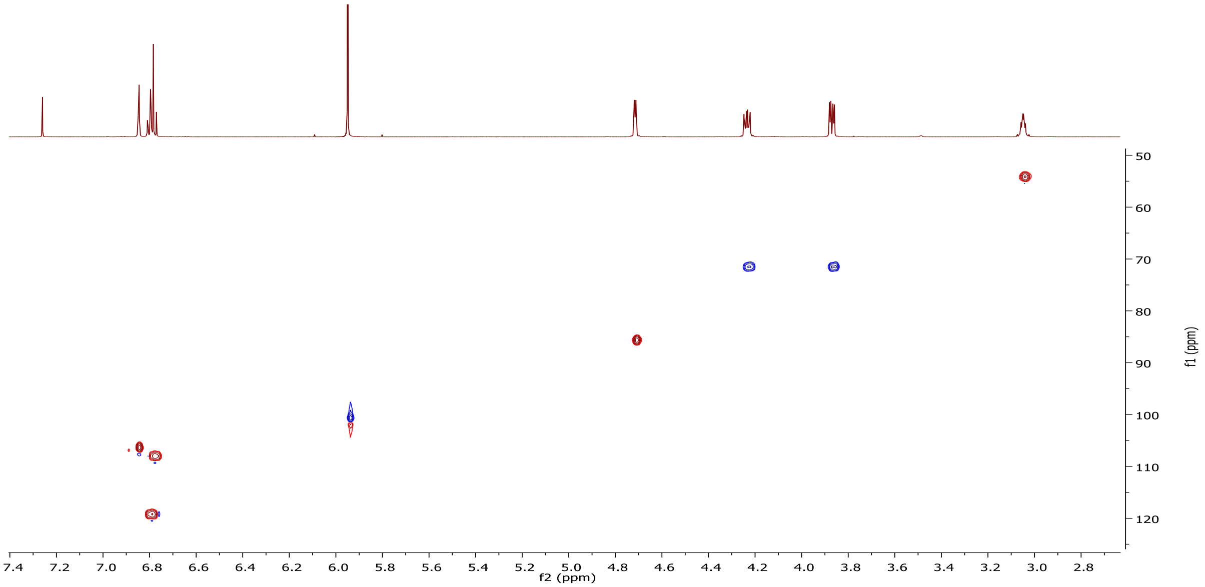

Supplementary Figure 2: c) 2D HSQC-DEPT NMR spectra of sesamin in $CDCl_3$.

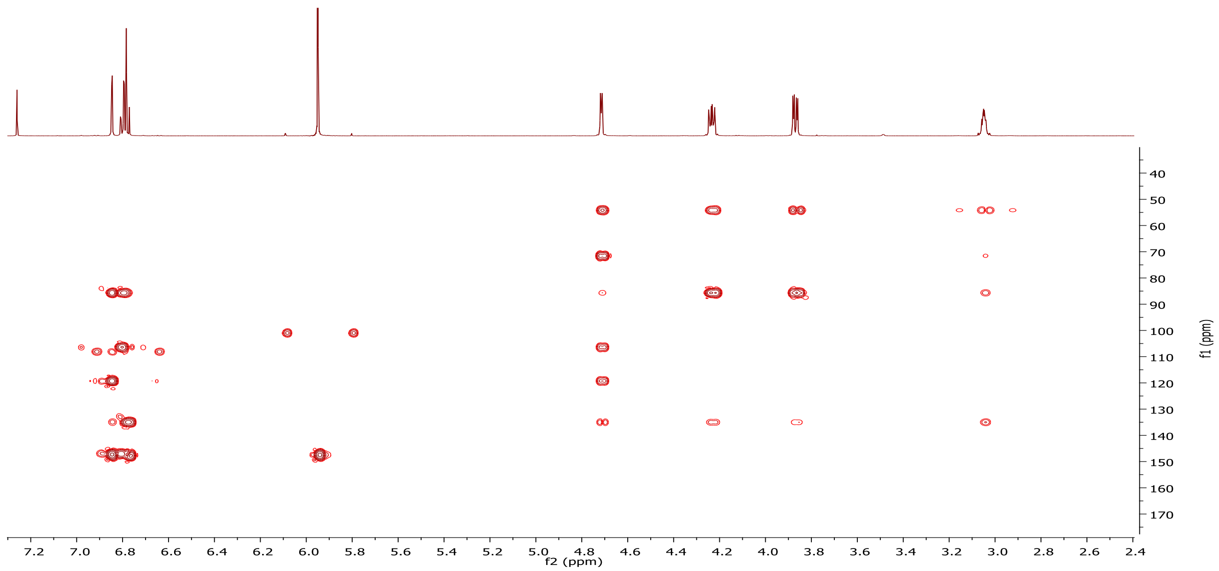

Supplementary Figure 2: d) 2D HMBC NMR spectra of sesamin in $CDCl_3$.

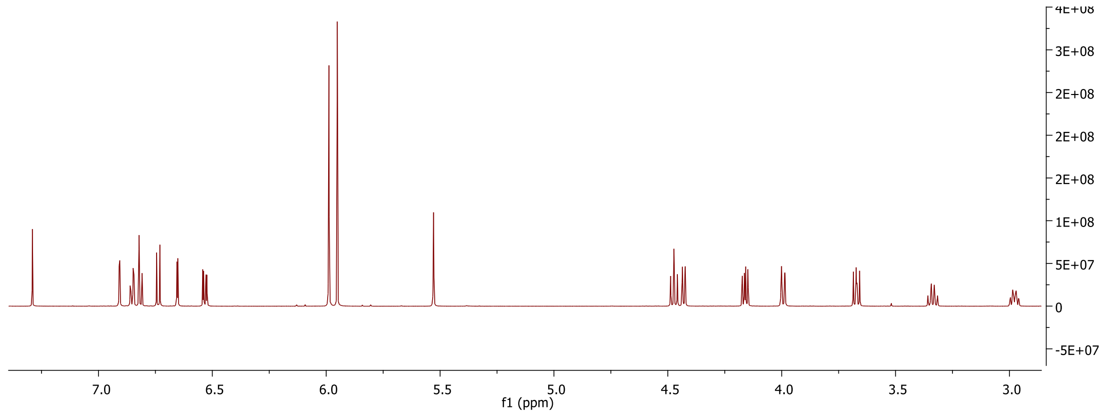

Supplementary Figure 3: a) ^{1}H NMR spectra of sesamolin in $CDCl_3$.

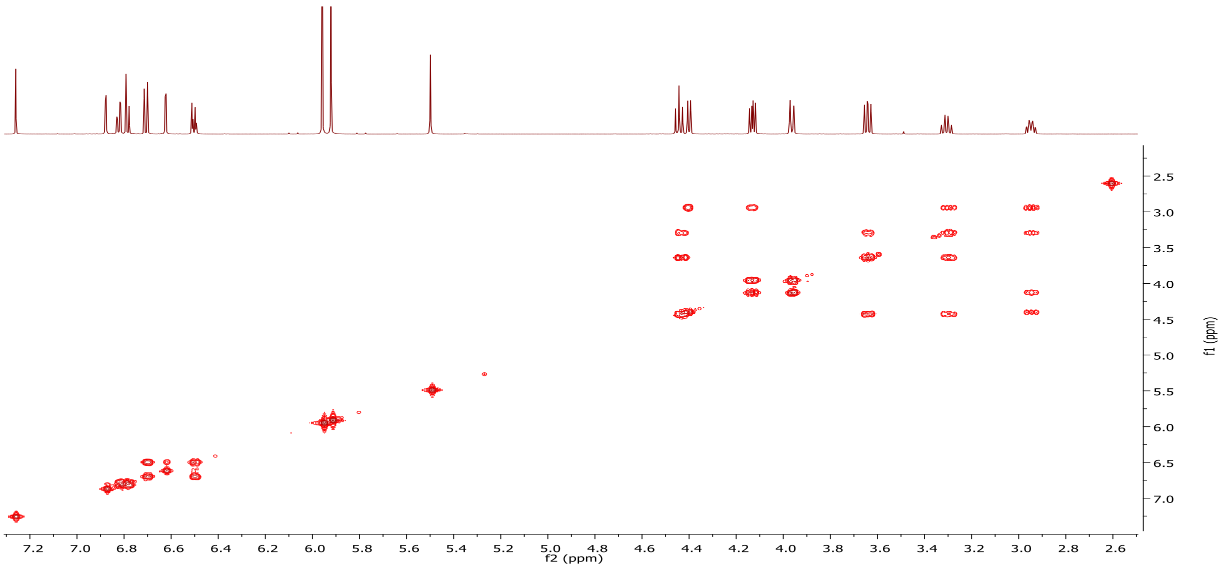

Supplementary Figure 3: b) 2D COSY NMR spectra of sesamolin in $CDCl_3$.

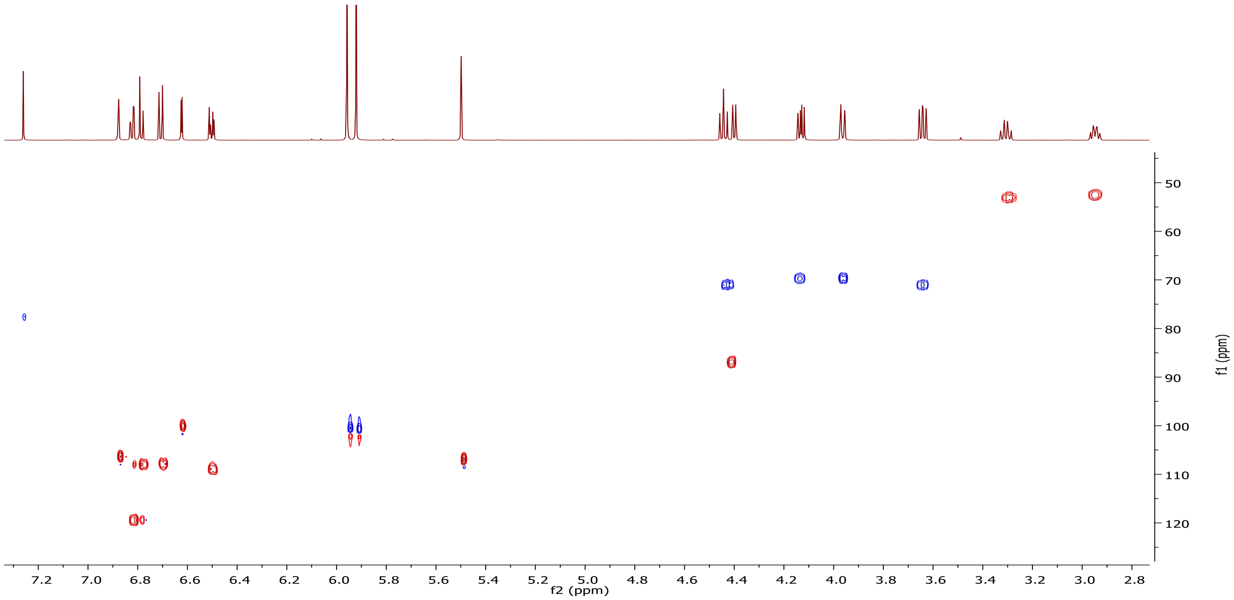

Supplementary Figure 3: c) 2D HSQC-DEPT NMR spectra of sesamolin in $CDCl_3$.

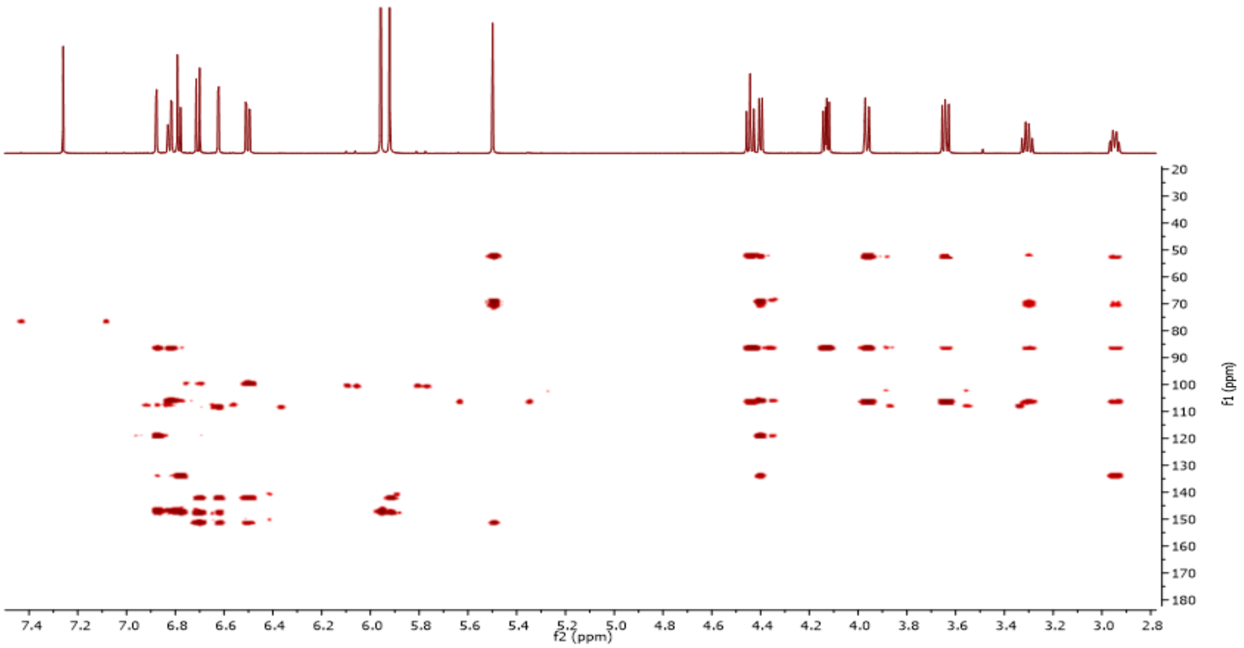

Supplementary Figure 3: d) 2D HMBC NMR spectra of sesamolin in $CDCl_3$.

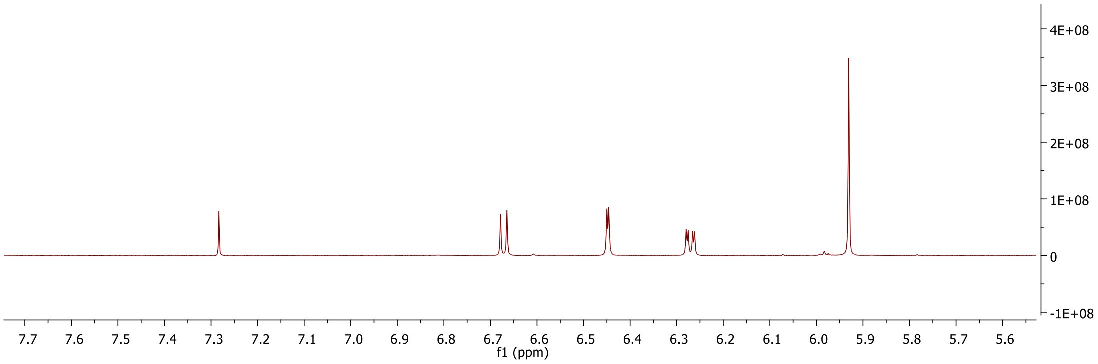

Supplementary Figure 4: ^{1}H NMR spectra of sesamol in $CDCl_3$.

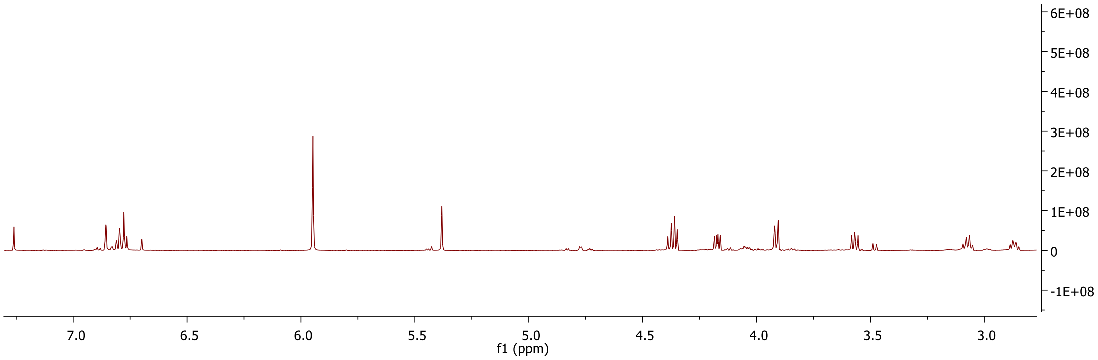

Supplementary Figure 5: a) ^{1}H NMR spectra of samin in $CDCl_3$.

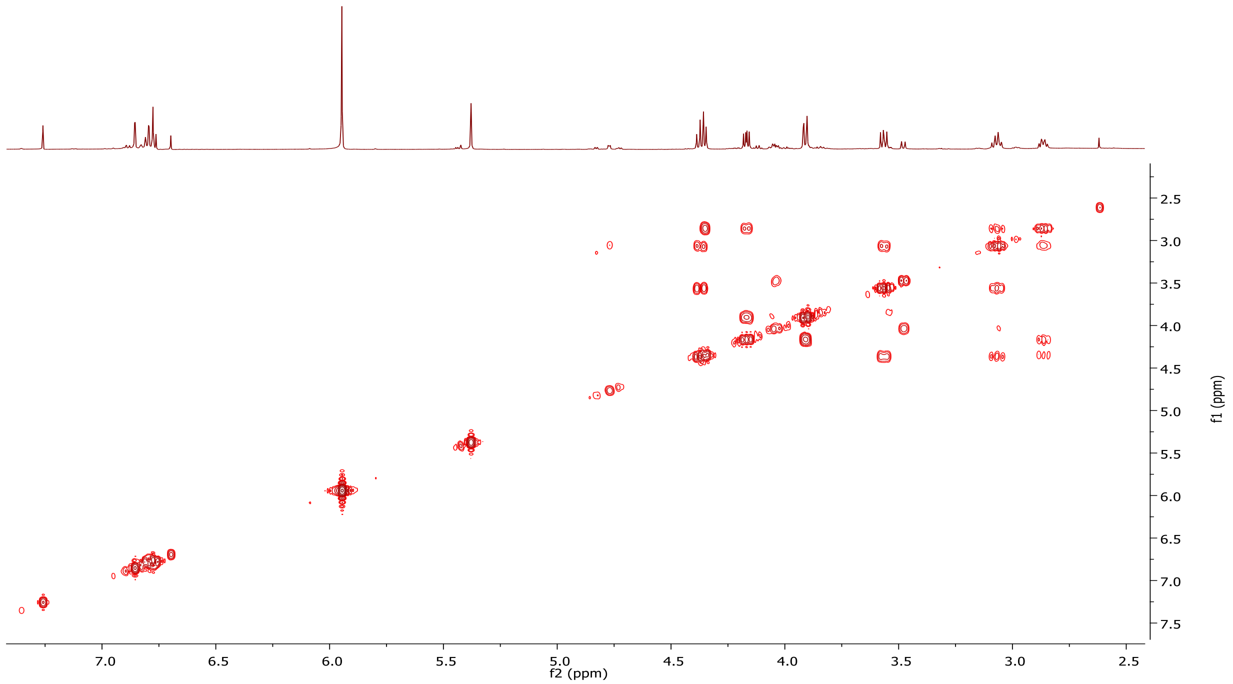

Supplementary Figure 5: b) 2D COSY NMR spectra of samin in $CDCl_3$.

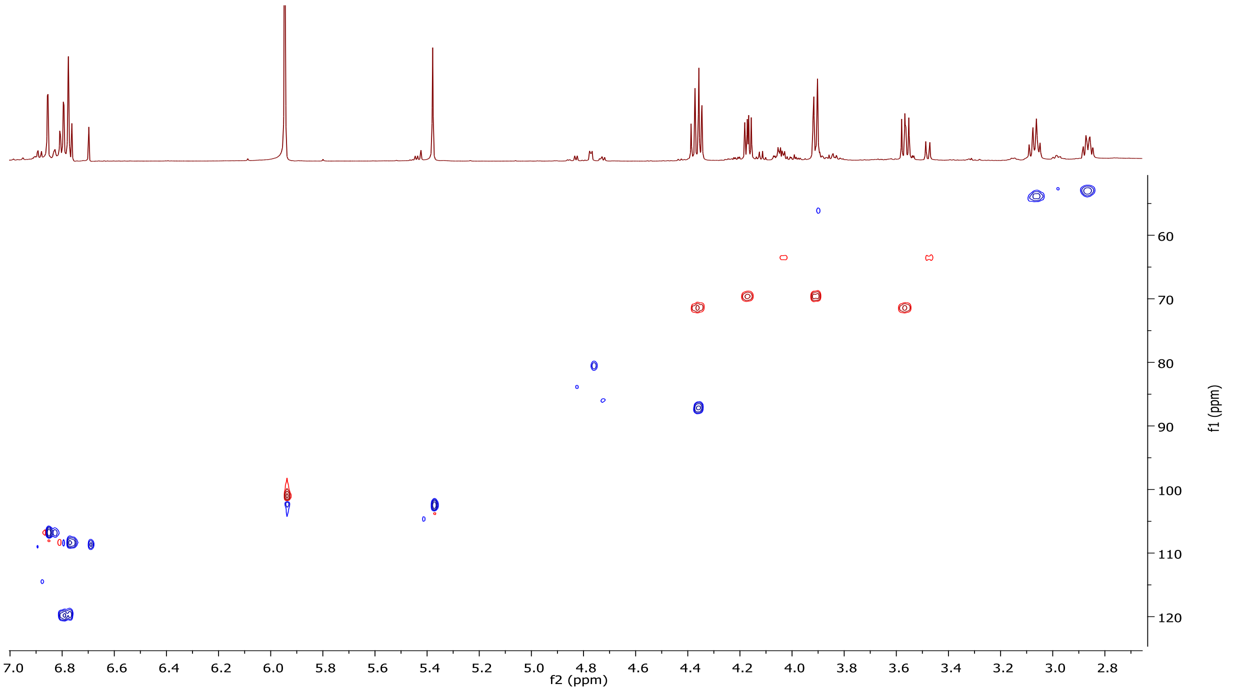

Supplementary Figure 5: c) 2D HSQC-DEPT NMR spectra of samin in $CDCl_3$.

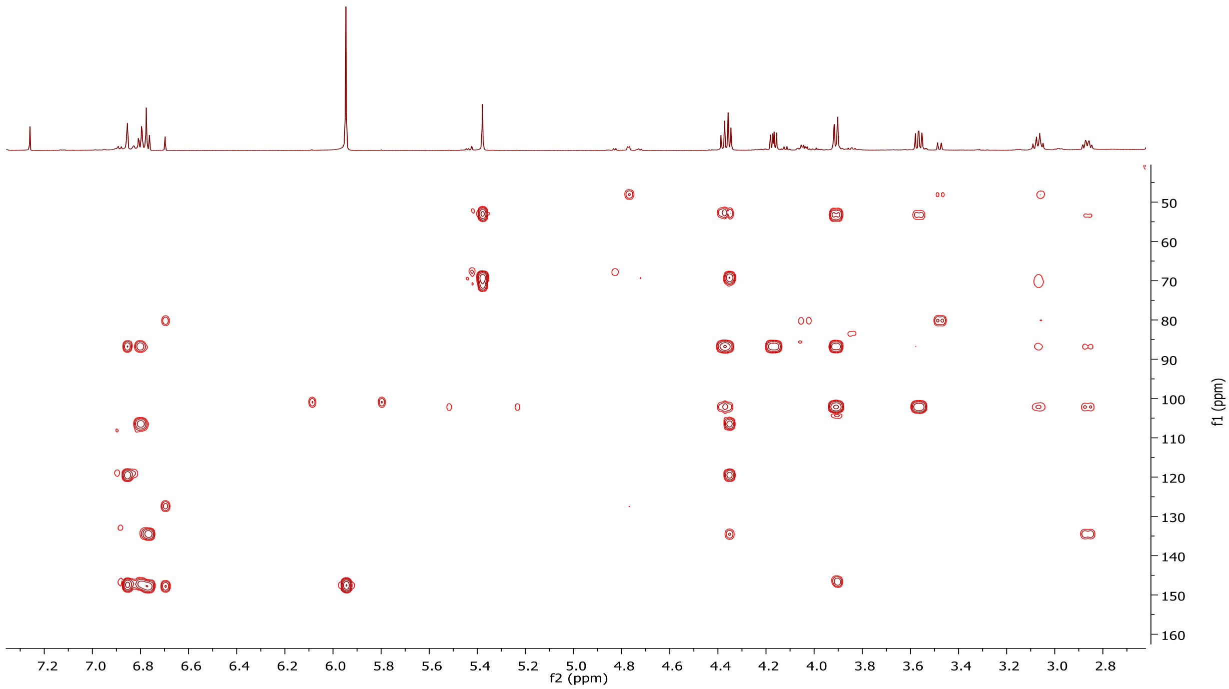

Supplementary Figure 5: d) 2D HMBC NMR spectra of samin in $CDCl_3$.

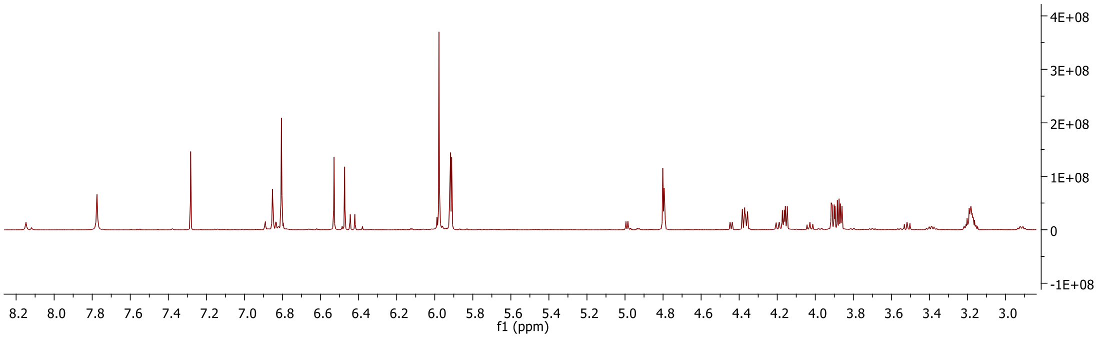

Supplementary Figure 6: a) ^{1}H NMR spectra of sesaminol-episesaminol in $CDCl_3$.

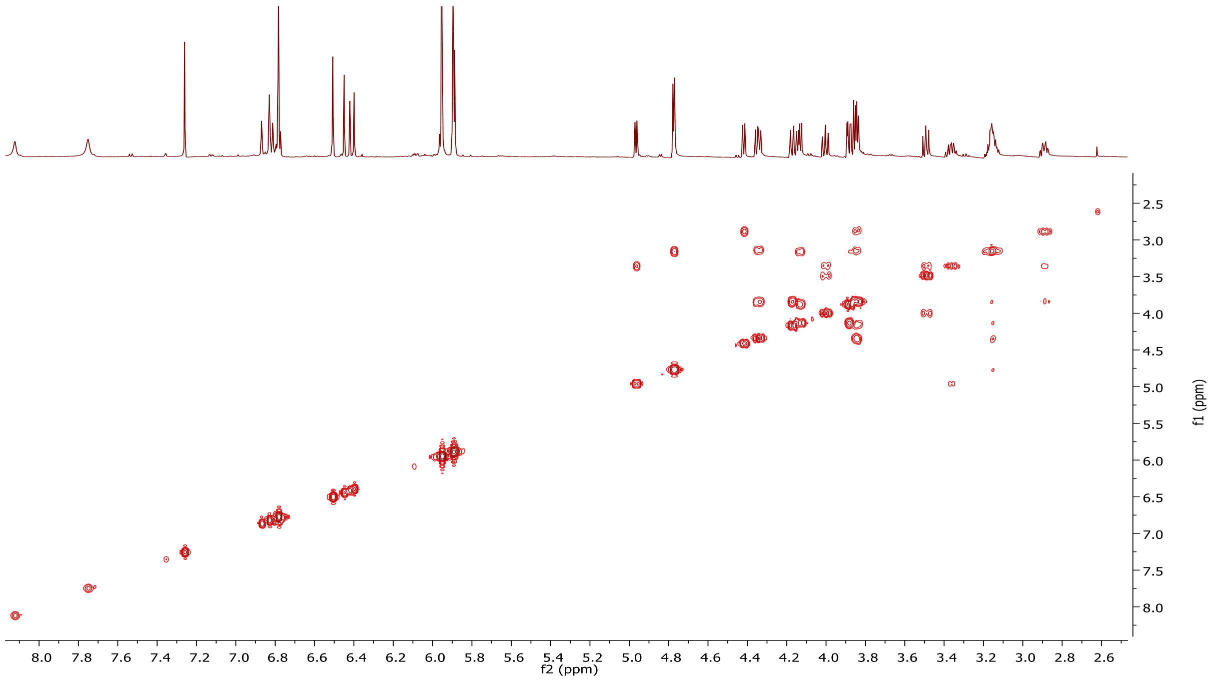

Supplementary Figure 6: b) 2D COSY NMR spectra of sesaminol-episesaminol in $CDCl_3$.

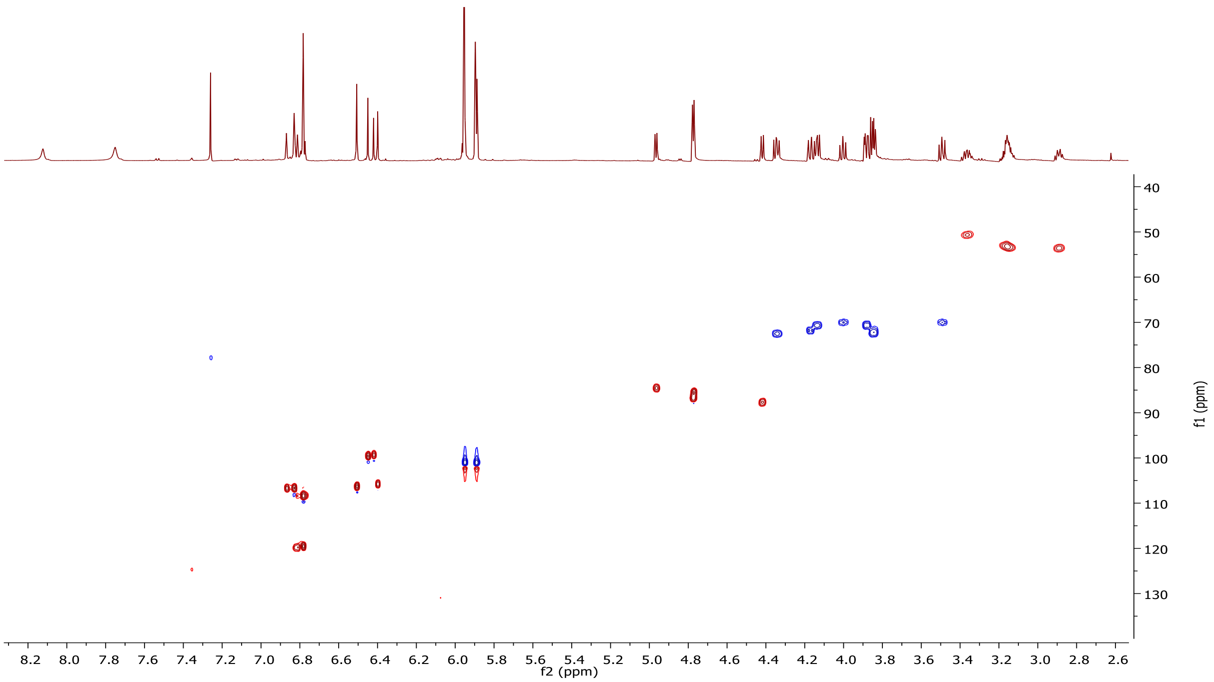

Supplementary Figure 6: c) 2D HSQC-DEPT NMR spectra of sesaminol-episesaminol in $CDCl_3$.

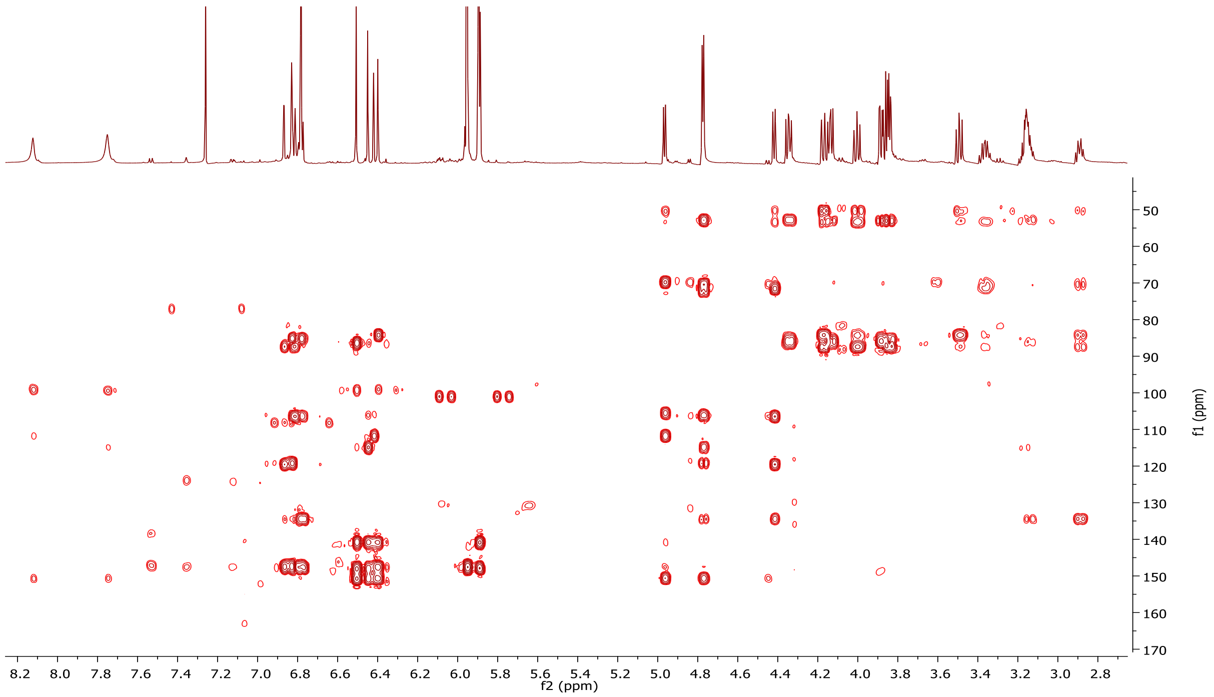

Supplementary Figure 6: d) 2D HMBC NMR spectra of sesaminol-episesaminol in $CDCl_3$.

1.2 Supplementary Tables

Supplementary Table 1: Solubility of SO extract and separation of biphasic CPC solvent system.

System	n-Hex	n-Hept	EtOAc	AcN	EtOH	MeOH	H_2O	Solubility of SO extract*	Separation of two phases**
CS1	4	-	1	-	2	-	3	++	+++
CS2	3	-	2	-	2	-	3	++	+++
CS3	2	-	3	-	2	-	3	+++	+++
CS4	1	-	4	-	2	-	3	+++	+++
CS5	4	-	1	-	3	-	2	+++	+++
CS6	3	-	2	-	3	-	2	+++	+++
CS7	2	-	3	-	3	-	2	+++	+++
CS8	1	-	4	-	3	-	2	+++	+++
CS9	-	2	3	-	-	3	2	++	-
CS10	-	2	3	2	-		3	+++	-
CS11	-	2	3	1	-	2	2	++	-
CS12	-	2	3	2	-	1	2	++	-
CS13	-	2	3	1	-	1	3	+++	+++
CS14	-	4	3	-	3	-	2	++	+++
CS15	-	4	3	1	2	-	2	++	+++
CS16	-	3	3	-	3	-	2	++	-
CS17	4	-	2,5	1,5	3	-	2	+++	+++

Supplementary Table 2: Liquid-liquid extraction system that tested on SO.

System	n-Hex	n-Hept	SO	MeOH	EtOH	H2O	AcN	H2O + 5% CH3COOH	H2O + 10% CH3COOH	BuOH	Comments
**ES1**	3	-	2	-	3	2	-	-	-	-	Creation of emulsion
**ES2**	3	-	2	-	2,5	2,5	-	-	-	-	Creation of emulsion
**ES3**	3	-	2	3	-	2	-	-	-	-	Creation of emulsion
**ES4**	3	-	2	3,5	-	1,5	-	-	-	-	Creation of emulsion
**ES5**	3	-	2	4	-	1	-	-	-	-	Creation of emulsion
**ES6**	4	-	1	3	-	2	-	-	-	-	Creation of emulsion
**ES7**	3	-	2	-	3	-	-	2	-	-	Creation of emulsion
**ES8**	3	-	2	-	3	-	-	-	2	-	Creation of emulsion
**ES9**	2	-	1	1	-	-	2	-	-	-	Creation of emulsion
**ES10**	3	-	2	1	-	-	4	-	-	-	Creation of emulsion
**ES11**	3	-	2	-	-	-	3	-	-	2	Separation in less than 1 min
**ES12**	3	-	1	-	-	-	4	-	-	1	Separation in less than 1 min
**ES13**	-	3	2	-	-	-	3	-	-	2	Separation in less than 1 min
**ES14**	-	3	1	-	-	-	4	-	-	1	Separation in less than 1 min
**ES15**	-	-	1	-	-	-	1	-	-	-	Separation in less than 45 sec
**ES16**	-	-	1	1	-	-	-	-	-	-	Separation in less than 45 sec
**ES17**	-	-	1	-	1	-	-	-	-	-	Separation in less than 45 sec

Supplementary Table 3: Partition coefficient of sesamin and sesamolin in system ES11, ES12, ES13 and ES14.

Extraction systems	Distribution coefficient of sesamin ($K=C_{up}/C_{low}$)	Distribution coefficient of sesamolin ($K=C_{up}/C_{low}$)
ES11	0,90	0,87
ES12	0,27	0,16
ES13	0,98	1,00
ES14	0,26	0,15

Supplementary Table 4: Area on HPLC chromatographs of sesamin and sesamolin in each of triple extraction for solvent systems ES15-ES17.

Extractions	Area of sesamin's peak	Area of sesamolin's peak
ES15 1th	13475090	8538380
ES15 2th	6255833	2946539
ES15 3th	2222615	945313
ES16 1th	6063140	2441181
ES16 2th	4429955	1837144
ES16 3th	3785633	1610077
ES17 1th	2177219	2177219
ES17 2th	1980610	1980610
ES17 3th	1547120	1547120

Supplementary Table 5. HPLC-HRMS (orbitrap) data of sesame oil total liquid-liquid extraction sample.

Rt	Experimental mass	Theoretical mass	Delta (ppm)	Molecular formula	RDB	ESI	Comments
**8.76**	137.0244	137.0246	1.625	$C_7 H_4 O_3$	5.5	-	sesamol
**10.32**	249.0767	249.0768	-0.5135	$C_{13}H_{14}O_5$	7.5	-	samin
**10.55**	371.1126	371.1125	0.2991	$C_{20}H_{18}O_7$	11.5	+	Sesaminol/episesaminol
**11.88**	371.1142	371.1125	4.5752	$C_{20}H_{18}O_7$	11.5	+	Sesamolin
**12.88**	355.1176	355.117	0.0859	$C_{20}H_{18}O_6$	11.5	+	sesamin
**20.89**	279.2325	279.2319	2.1332	$C_{18}H_{32}O_2$	3.5	-	Linoleic acid

Supplementary Table 6. NMR signals of sesamin, sesamolin, sesamol, samin, sesaminol and episesaminol.

Protons	Sesamin	sesamolin	sesamol	samin		sesaminol		Episesaminol	
	^{1}H (ppm)	^{1}H (ppm)	^{1}H (ppm)	^{1}H (ppm)	^{13}C (ppm)	^{1}H (ppm)	^{13}C (ppm)	^{1}H (ppm)	^{13}C (ppm)
1	**3.05**, m	**2.95**, m		**2.86**, m	**53.14**	**3.16**, m	**53.3**	**2,88**, m	**53.6**
2	**4.71**, d<br>J= 4.5 Hz	**4.40**, d<br>J=7.1 Hz		**4.35**, d<br>J=6.8 Hz	**87.34**	**4.77**, m	**85.5**	4.42, d<br>J=7.0 Hz	**87.8**
3			**6.43**, d<br>J=2.3 Hz						
4a	**4.23**, dd<br>J= 9.2, 6.9 Hz	**4.44**, t<br>J=9.0 Hz		**4.38**, t<br>J=9.1 Hz	**71.64**	**4.34**, dd<br>J=9.4, 7.5 Hz	**72.6**	**4.17**, brd<br>J=9.5 Hz	**71.9**
4b	**3.86**, dd<br>J= 9.2, 3.8 Hz	**3.64**, dd<br>J=9.2, 7.4 Hz		**3.57,** dd<br>J=9.1, 7.4 Hz	**71.65**	**3.85**, dd<br>J=9.4, 5.3 Hz	**72.6**	**3.85,** m	**71.9**
5	**3.05**, m	**3.31**, m	**6.25**, dd<br>J=8.4, 2.3 Hz	**3.07**, m	**54.08**	**3.16**, m	**53.3**	**3.36**, m	**50.8**
6	**4.71**, d<br>J=4.5		**6.65**, d<br>J=8.4 Hz	**5.38**, bs	**102.6**	**4.78**, bs	**86.7**	**4.96**, d<br>J=5.0 Hz	**84.6**
8a	**4.23**, dd<br>J= 9.2, 6.9 Hz	**4.13**, dd<br>J=9.2, 5.9 Hz		**4.17**, dd<br>J=9.1, 5.9 Hz	**96.84**	**4.14**, dd<br>J=9.4, 2.4 Hz	**70.6**	**4.00**, d<br>J=9.0 Hz	**70.4**
8b	**3.86**, dd<br>J= 9.2, 3.8 Hz	**3.96**, dd<br>J=9.1, 0.8 Hz		**3.91**, bd<br>J=8.5 Hz	**96.84**	**3.88**, dd<br>J=9.4, 2.4 Hz	**70.6**	**3.49**, d<br>J=9.0 Hz	**70.4**
2’	**6.85**, d<br>J=1.5 Hz	**6.88**, d<br>J=1.7 Hz		**6.86**, d<br>J=1.5 Hz	**106.85**	**6.83**, brs	**106.7**	6.86, brs	**106.8**
5’	**6.77**, d<br>J=8.1 Hz	**6.78**, d<br>J=8.0 Hz		**6.77**, d<br>J=7.9 Hz	**108.44**	**6.78**, brs	**108.4**	**6.78**, d<br>J=8.1 Hz	**108.4**
6’	**6.80**, dd<br>J=8.1 Hz	**6.82**, dd<br>J=8.0, 1.7 Hz		**6.80**, dd<br>J=7.9, 1.5 Hz	**119.91**	**6.78**, brs	**119.5**	**6.81**, brd<br>J=8.1 Hz	**119.9**
2’’	**6.85**, d<br>J=1.5 Hz	**6.62**, d<br>J=2.3 Hz				**6.0**, s	**106.4**	**6.40**, s	**105.9**
5’’	**6.77**, d<br>J=8.1 Hz	**6.71**, d<br>J=8.5 Hz				**6.45**, s	**99.5**	**6.42**, s	**99.4**
6’’	**6.80**, dd<br>J=8.1 Hz	**6.82**, dd<br>J=8.5, 2.3 Hz							
-O-CH_2-O-	**5.95**, s		**5.91**, s	**5.95**, s	**101.15**				
-O-CH_2-O- a		**5.92**, s				**5.95**, s	**101.0**	**5.95**, s	**101.0**
-O-CH_2-O- b		**5.96**, s				**5.89**, s	**101.1**	**5.89**, s	**101.1**
-OH						**7.75,** brs	**150.8**	**8.12**, brs	**150.9**

1.3 Supplementary Diagrams

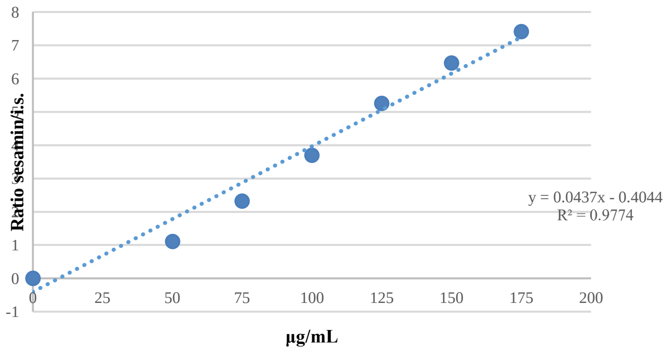

Supplementary Diagram 1: Quantification curve of sesamin using internal standard (i.s.).

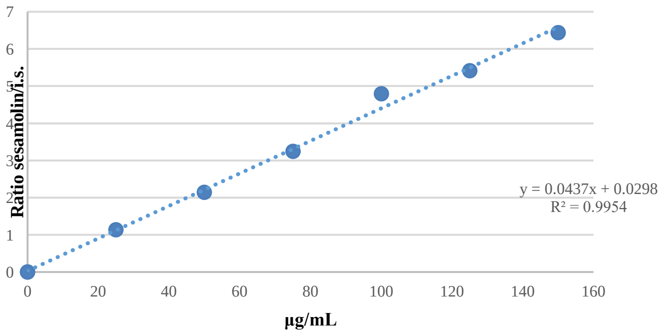

Supplementary Diagram 2: Quantification curve of sesamolin using internal standard (i.s.).
